# Effectiveness of interventions to address the negative health outcomes of informal caregiving to older adults: protocol for an umbrella review

**DOI:** 10.1136/bmjopen-2021-053117

**Published:** 2021-11-09

**Authors:** Amaia Calderón-Larrañaga, Mariam Kirvalidze, Lena Dahlberg, Lawrence B Sacco, Lucas Morin

**Affiliations:** 1 Aging Research Center, Department of Neurobiology, Care Sciences and Society, Karolinska Institutet, Solna, Sweden; 2 School of Health and Welfare, Dalarna University, Falun, Sweden; 3 Stress Research Institute, Department of Psychology, Stockholm University, Stockholm, Sweden; 4 Inserm CIC 1431, University Hospital of Besançon, Besançon, France; 5 Department of Medical Epidemiology and Biostatistics, Karolinska Institutet, Solna, Sweden

**Keywords:** public health, health policy, qualitative research, clinical trials

## Abstract

**Introduction:**

Informal (unpaid) caregivers play an essential role in caring for older people, whose care needs are often not fully met by formal services. While providing informal care may be a positive experience, it can also exert a considerable strain on caregivers’ physical and mental health. How to best support the needs of informal caregivers remains largely debated. This umbrella review (review of systematic reviews) aims to evaluate (1) whether effective interventions can mitigate the negative health outcomes of informal caregiving, (2) whether certain types of interventions are more effective than others, (3) whether effectiveness of interventions depends on caregiver/receiver, context or implementation characteristics and (4) how these interventions are perceived in terms of acceptability, feasibility and added value.

**Methods and analysis:**

We will include systematic reviews of primary studies focusing on the effectiveness of interventions (public or private, unifaceted or multifaceted, delivered by health or social care professionals or volunteers) aimed at reducing the impact of caregiving on caregivers’ physical or mental health. This will also include quantitative and qualitative syntheses of implementation studies. The literature search will include the following databases: Medline, CINAHL, PsycINFO and Web of Science. A key informant-guided search of grey literature will be performed. Quality appraisal will be conducted with the AMSTAR-2 checklist for quantitative reviews and with an *ad hoc* checklist for qualitative syntheses. Narrative and tabular summaries of extracted data will be produced, and framework synthesis will be employed for weaving together evidence from quantitative studies in effectiveness reviews with findings on implementation from qualitative studies.

**Ethics and dissemination:**

This umbrella review will use data from secondary sources and will not involve interactions with study participants; it is thus exempt from ethical approval. Results will be presented at international conferences and will be published in a peer-reviewed journal.

**PROSPERO registration number:**

CRD42021252841.

**Strengths and limitations of this study**

- The umbrella review methodology will enable us to perform higher level analysis by synthesising the evidence from existing systematic reviews addressing the effectiveness of interventions to mitigate the negative health consequences of informal caregiving.
- It will be possible to include both quantitative reviews on effectiveness together with qualitative syntheses exploring complex aspects related to acceptability, feasibility and added value.
- Due to heterogeneity in review designs, findings will not be directly comparable, and synthesis might be confined to a narrative output.
- More recent primary studies on new interventions may not be captured, as they may not yet have been included in systematic reviews.

## Introduction

Informal caregiving to older adults has become a major societal challenge globally. Ageing demographics and ageing-in-place-policies have led to an increased number of older people living the last part of their lives at home.^1^ A considerable fraction of these individuals has complex care needs—characterised by multiple conditions, cognitive and physical impairments—and require substantial support, beyond that provided by formal care services.^2^ These demands are especially intense in times of public health crisis, such as the COVID-19 pandemic, during which many older people have been asked to self-isolate in their homes.^3^

The number of informal (unpaid) caregivers has grown fast over the last years, contributing to the majority of the care received by those aged over 50 in most European countries,^4^ and it is expected that the number of people taking up the caregiver role will increase in the next years.^5^ Despite the noticeable increase in the use of privately purchased care services, these mostly play a marginal role.^6^ Instead, a major trend towards the *informalisation* of social care has been observed since the 1990s.^7 8^ While providing informal care may be lived as a positive experience,^9^ it can also be distressing and have negative health consequences.^10^ A state of subjective burden is characterised by fatigue and stress in the caregiver,^11^ and by a higher risk of institutionalisation and psychiatric symptoms in the care receiver.^12^

The literature regarding the management of caregiver burden is evolving rapidly and an increasing number of primary studies and systematic reviews assessing the effectiveness of different interventions have been published.^13–15^ Yet, we still do not know how best to support the diverse needs of informal caregivers throughout the course of their ‘invisible journey’ in caregiving.^16 17^ This is mainly due to a lack of nuance in understanding how their needs may differ depending on their experience of caregiving. Several factors play an essential role in shaping this intimate and highly personal experience. The development of the *stress process*^18^ among caregivers is influenced by the intensity of care demands, the length and current stage of the caregiving journey, the existence of other sources of strain, the caregivers’ coping style and the density of their social network.^19^ The consequences of informal caregiving also vary depending on the relationship between the care receiver and the caregiver, and on the acute or chronic nature of the care receiver’s condition.^20^

Therefore, in designing caregiver interventions, it is essential to distinguish between elements that might be broadly applicable to all informal caregivers at large and elements that are specific to certain caregivers, care receivers’ health problems and contexts of care.^21^ Accordingly, a systematic review of 31 randomised controlled trials in the context of dementia and Alzheimer’s disease found insufficient evidence to endorse the use of most interventions but noted larger trials that employed tailored interventions had significant effects on at least one outcome.^22^ This exemplifies the limitation of taking a ‘blanket approach’ to designing interventions for caregivers at large, as these might fail to recognise significant effects on subsets of the caregiver population, thereby underestimating the wide heterogeneity in informal care provision and in the circumstances of caregivers.

Systematic reviews and meta-analyses of caregiving interventions often consider study populations as homogeneous, which masks contextual factors such as culture, financial resources or health literacy that impact caregivers’ risk of negative physical and mental health outcomes.^23^ This one-size-fits-all approach has been shown to be inadequate, but remains common in informal caregiving research.^24^ We believe an umbrella review (i.e. a review of systematic reviews)^25^ is a necessary step that will provide a broader picture of the effectiveness of different types of support services targeting different pools of informal caregivers. This requires combining an ‘aggregative approach’, whereby quantitative reviews are used to answer well-defined questions based on empirical observations, together with more ‘configurative approaches’ drawing both on quantitative and also qualitative reviews.^26^

The aim of this umbrella review is to synthesise the evidence from existing systematic reviews evaluating the effectiveness of interventions to address the negative health consequences of informal caregiving. In particular, the following research questions will be addressed: (1) Are there effective interventions to prevent and reduce the negative health consequences of informal caregiving? (2) Are certain types of interventions more effective than others? (3) Is there evidence that the effectiveness of interventions depends on caregiver, care receiver, care context and implementation characteristics? (4) How are the proposed interventions experienced by caregivers in terms of their acceptability, feasibility and added value?

To date, there are a few umbrella reviews or meta-reviews published on the topic of caregiver support. However, these reviews either include only one type of support intervention and/or disease,^27–29^ or are too broad in nature to capture differences between caregiver groups.^30^ This is the first umbrella review with a focus on caregivers of older populations, exploring objective physical and mental health-related outcomes and employing an integrative approach in order to complement quantitative effectiveness reviews with qualitative evidence on how caregivers experience support interventions.

## Methods and analysis

### Study design

We will conduct an umbrella review, defined as a ‘review of existing systematic reviews and meta-analyses’.^25^ Umbrella reviews focus on broad conditions or problems (e.g., interventions to mitigate the negative health consequences of informal caregiving) for which there is a rich and high-quality evidence base. The bird’s-eye view provided by an umbrella review is well suited to examine whether the evidence base around a given research question is consistent or contradictory, and to explore the underlying reasons in detail.^25^ Furthermore, it will enable us to assess whether review authors who worked independently from each other but addressed similar research questions observed similar results and drew similar conclusions. This umbrella review will be reported in accordance with the Preferred Reporting Items for Overviews of Reviews (PRIOR) statement.^31^ The review is anticipated to be conducted in the period of 1 April 2021– 31 May 2022.

### Target population

We will include systematic reviews of quantitative, qualitative or mixed-methods primary studies with or without meta-analysis focusing on interventions to address the negative health consequences of informal caregiving for informal caregivers of older adults. The full list of inclusion and exclusion criteria developed for this umbrella review is provided in box 1.

Box 1 **Inclusion and exclusion criteria for quantitative and qualitative reviews**

**Inclusion criteria**

*Publication type, date and language*

- Reviews published in a peer-reviewed journal OR as a report done or mandated by an official health agency (*e.g., Health Technology Assessment agency*).
- Reviews published between 1 January 2000 and 26 March 2021.
- Reviews published in English, Swedish, Spanish, French, Italian or German.

*Study design*

- *For quantitative reviews*: reviews including a reproducible, systematic search strategy AND clearly defined inclusion/exclusion criteria AND risk of bias assessment for all included primary studies.
- *For qualitative reviews*: reviews including a reproducible, systematic search strategy AND defined inclusion/exclusion criteria.

*Population*

- Reviews concerning informal caregivers (*i.e., people who regularly provide unpaid care to a family member, friend or neighbour*) of older people OR of persons presenting with ageing-related disease (*eg, dementia, stroke, Parkinson’s disease, cancer, heart failure, multimorbidity, frailty*).

*Intervention*

- Reviews focusing on interventions and assessing either their effectiveness (*for quantitative reviews*) or their implementation and/or the lived experience of the target population (*for qualitative reviews*).

*Outcome*

- Reviews including physical or mental health-related outcomes of informal caregivers, including health-related quality of life.

**Exclusion criteria**

- Reviews of interventions focusing *exclusively* on care receivers as the target population.
- Reviews focusing *exclusively* on interventions for caregivers of young populations.
- Reviews measuring *exclusively* non-health-related outcomes, such as caregiver burden, stress/strain, work or financial status, family relations, breakdown of informal care.
- Reviews focusing exclusively on end-of-life interventions or interventions implemented in the context of formal care institutions, including those addressing the transition from home to care homes.

For the purpose of this umbrella review, informal caregivers will be defined as any relatives, partners, friends or neighbours who have a significant personal relationship with, and provide a broad range of unpaid assistance for, older persons with a chronic or disabling condition.^32^ Some have argued that the term ‘family caregiver’ should be preferred over ‘informal caregiver’ on the ground that caregivers may find the latter invalidating and belittling.^33^ Although we acknowledge the importance of terminology around this complex issue, this umbrella review will use the term ‘informal caregiving’ to explicitly include non-familial caregivers in its scope. Older care receivers will be defined by chronological age or as having an age-related disease or syndrome such as dementia, cancer, stroke, Parkinson’s disease, heart failure, multimorbidity and/or frailty. This corresponds with the broad range of caregiver experiences and needs that healthcare and social care providers tend to encounter. We will exclude reviews focusing exclusively on interventions directed towards care receivers, as well as those focusing on interventions implemented in the context of institutional care (addressing, for instance, the transition from home to care homes, or the situation of disabled people ageing in residential facilities). Reviews of interventions specific to the context of end-of-life care will also be excluded, since caregiving approaches at this stage of the illness trajectory warrant a separate review and discussion.^34^

### Interventions, comparators and outcomes

Systematic reviews will be included if they provide synthesised results about the effectiveness (i.e., the extent to which an intervention, when used appropriately, achieves the intended effect)^35^ and implementation (i.e., how and why interventions work within real-world conditions)^36^ of interventions aimed at preventing and reducing negative health outcomes in the caregivers. We will examine all types of interventions (public or private), whether they are unifaceted or multifaceted interventions, and delivered by all types of healthcare or social care professionals or volunteers. The typology developed by Gaugler *et al*^37^ will be adapted to categorise intervention types: skill building, psychosocial support, education, cognitive and behavioural approaches, respite, care/case management and relaxation/physical activity.

In those reviews where a comparator is required, any comparator(s) tested will be considered: intervention versus control (no intervention, usual care, placebo or other control, or another intervention) as defined by the original reviews. We will include different caregiver-related health outcomes, since we want to give an overview of the different outcome measures used in the included reviews. This entails both physical and mental diseases, syndromes and symptoms, and also health-related quality of life and self-perceived health status. However, we will exclude reviews measuring exclusively non-health-related outcomes, such as caregiver burden, family relations, work and financial status or stress/strain in the caregiver.

### Publication type, date and language

Quantitative, qualitative or mixed-methods reviews published in a peer-reviewed journal or as a report done or mandated by an official health agency (e.g., Health Technology Assessment agency) will be included. Even if the first reviews on caregiving intervention research were published in the 1990s, we will focus on reviews published since 2000 in an attempt to capture studies taking place in the context of recent social changes and advancements in caregiving intervention research. We will restrict the inclusion to reviews published in languages spoken by our research team members: English, Swedish, Spanish, French, Italian and German.

### Data sources and search strategy

An iterative search strategy for bibliometric databases and grey literature will be developed to look for peer-reviewed systematic reviews evaluating interventions addressing the negative health consequences in the informal caregiver in all care settings, including quantitative and/or qualitative syntheses of implementation studies and process evaluations alongside trials testing such interventions. Therefore, the search for this umbrella review will aim to identify all research syntheses relevant to our four research questions. The search strategy will be guided by a librarian, comprehensively reported, and the detailed search filters employed will be presented sequentially in the single appendix for all the databases that were searched and listed along with the search dates. The literature search will be undertaken using the following biomedical and sociological citation databases: Medline, CINAHL, PsycINFO and Web of Science. We will use *CoCites* (https://www.cocites.com/) citation-based search tool to widen the net and retrieve articles that cite eligible systematic reviews.^38^

The preliminary search strategy is detailed in table 1. This search strategy has been developed by the research team with the guidance of experienced health sciences librarians to estimate the number of potentially relevant systematic reviews. It combines free-text words with controlled vocabulary terms (such as Medical Subject Headings (MeSH) terms/Thesaurus terms/CINAHL Subject Headings). Additional searches will be developed for syntheses of effectiveness, implementation or process evaluations published or mandated by official health agencies. We also expect a substantial number of such reports meeting our criteria not to be necessarily published in peer-reviewed journals. Thus, we will contact the first and last authors of selected reviews to retrieve grey literature that may otherwise have been missed. Finally, we will perform a manual search of the reference lists of relevant reviews.

**Table 1:** Search strategy developed by the research team to estimate the sample size

#	Searches
1	exp Aged/
2	exp Aging/
3	Frailty/
4	(advanced age or aged or ageing or aging or elder* or frail* or geriatr* or gerontolog* or late* life or old age or old* adult* or old* client* or old* individual* or old* man or old* men or old* patient* or old* people or old* person* or old* population* or old* woman or old* women or oldest old or retired or senior*).ti, ab, kf.
5	Dementia/
6	Alzheimer Disease/
7	(dementia* or alzheimer*).ti, ab, kf.
8	exp Neoplasms/
9	(neoplasm* or cancer*).ti, ab, kf.
10	exp Stroke/
11	stroke.ti, ab, kf.
12	Parkinson disease/
13	parkinson*.ti, ab, kf.
14	Multimorbidity/
15	multimorbid*.ti, ab, kf.
16	exp Heart failure/
17	(heart failure or cardiac failure).ti, ab, kf.
18	or/1–17
19	Caregivers/
20	(caregiv* or care giv* or caretak* or care tak* or carer*).ti, ab, kf.
21	((family or informal or unpaid) adj3 (care or caring)).ti, ab, kf.
22	19 or 20 or 21
23	(meta analysis or systematic review).pt.
24	review.ti.
25	systematic* review*.ab, kf.
26	(meta analy* or metaanaly* or meta stud* or meta interpretation* or meta ethnograph* or meta summar* or meta synthes* or meta narrative* or mixed research synthes*).ti, ab, kf.
27	((concept analy* or grounded theory) and review*).ti, ab, kf.
28	or/23–27
29	18 and 22 and 28
30	(english or swedish or spanish or french or italian or german).lg.
31	29 and 30
32	limit 31 to yr=‘2000 -Current”
33	limit 32 to (comment or congress or editorial or letter)
34	32 not 33

Field labels: exp/, exploded MeSH term; /, non-exploded MeSH term;.ti, ab, kf., title, abstract and author keywords;.pt., publication type; adjx, within x words, regardless of order; *, truncation of word for alternate endings.

Database(s): Ovid MEDLINE(R) and Epub Ahead of Print, In-Process, In-Data-Review and Other Non-Indexed Citations and Ovid MEDLINE(R) Daily 1946 to 26 March 2021.

### Study selection

Titles and abstracts of the citations will be screened by two independent reviewers by using the Covidence (https://www.covidence.org/) software developed by the Cochrane collaboration.^39^ Before screening, inclusion criteria will be tested in a pilot study. We will report any changes to the inclusion and exclusion criteria that result from the calibration exercise as deviations from the published protocol. Full texts of the relevant documents will be independently checked for eligibility by two independent reviewers using Covidence. Any dissent in abstract screening and full-text assessment will be resolved by discussion moderated by a third reviewer. Reviews excluded during the full-text assessment will be documented along with the reason for exclusion and presented using the Preferred Reporting Items for Systematic Reviews and Meta-Analyses (PRISMA) flowchart.^40^

### Methodological quality and risk of bias assessment

Critical appraisal of quantitative reviews will be conducted independently by two reviewers using the AMSTAR-2 checklist.^41^ The checklist categorises the quality of the reviews based on seven critical and nine non-critical domains.^41^ Based on the appraisal, the reviews will be grouped into critically low, low, moderate and high-quality categories. Qualitative reviews will be assessed by two reviewers using an ad hoc quality appraisal checklist (online supplemental appendix 1), adapted from the Joanna Briggs Institute Checklist for Systematic Reviews and Research Syntheses.^25^ The tool has been developed and piloted by all members of our multidisciplinary group. As far as possible, the quality of mixed-methods systematic reviews will be assessed using the above-mentioned tools for the quantitative and qualitative components, respectively. Any dissent in the process of quality assessment will be resolved through discussions moderated by a third reviewer.

10.1136/bmjopen-2021-053117.supp1 Supplementary data

### Data extraction

Data will be extracted independently by two reviewers according to the type of systematic review (i.e., systematic reviews of quantitative, qualitative studies and/or mixed-methods studies), using an ad hoc data extraction form that will be appended to the manuscript. This form will be calibrated during a pilot phase, with a purposive sample of five studies, to ensure that all relevant data are extracted in a consistent manner. Extraction will be limited to data reported in the reviews and will include broad categories of descriptive characteristics (such as number and list of primary studies, caregiver and care receiver demographics, context of care, type of interventions, etc.) and synthesised findings of the review (either meta-analysis or narrative findings). Clear indication of overlap of primary research studies in each of the included reviews will be presented, and significant overlap will be addressed to avoid double counting.

### Data synthesis

A narrative description of the included reviews will be provided, with reference to a detailed table of included review characteristics. This description will allow for contextualising the results in terms of the relevance and evidence base of included research syntheses for the umbrella review questions. Specific items or points of interest from individual reviews will also be highlighted, as described below.

Overall effect estimates from quantitative systematic reviews will be presented in tables, alongside a detailed description of the interventions assessed. In addition to the direction and magnitude of effects, number of reviews that inform each outcome, number of participants and statistical heterogeneity, factors related to the caregiver, care receiver and/or care context affecting the effectiveness of interventions will be identified whenever possible. This will attempt to answer research questions 1, 2 and 3, related to effectiveness, comparison of interventions, and factors affecting the effectiveness of interventions, respectively.

Evidence from syntheses of qualitative research will also be presented in tabular format. Results will be synthesised and illustrated by using verbatim replications from the source review where appropriate. Findings will also be described and interpreted in the research team’s own words. Caregivers’ experiences and views on barriers to and facilitators of interventions will be identified in terms of acceptability, feasibility and added value (research question 4). The diversity of caregivers, care receivers and/or care contexts will be considered as far as possible.

Framework synthesis methods will be employed for the integration of quantitative studies in effectiveness reviews with qualitative and process evaluation evidence. These methods are particularly suitable for capturing complexity of interventions in systematic reviews.^42^

**Figure 1:**
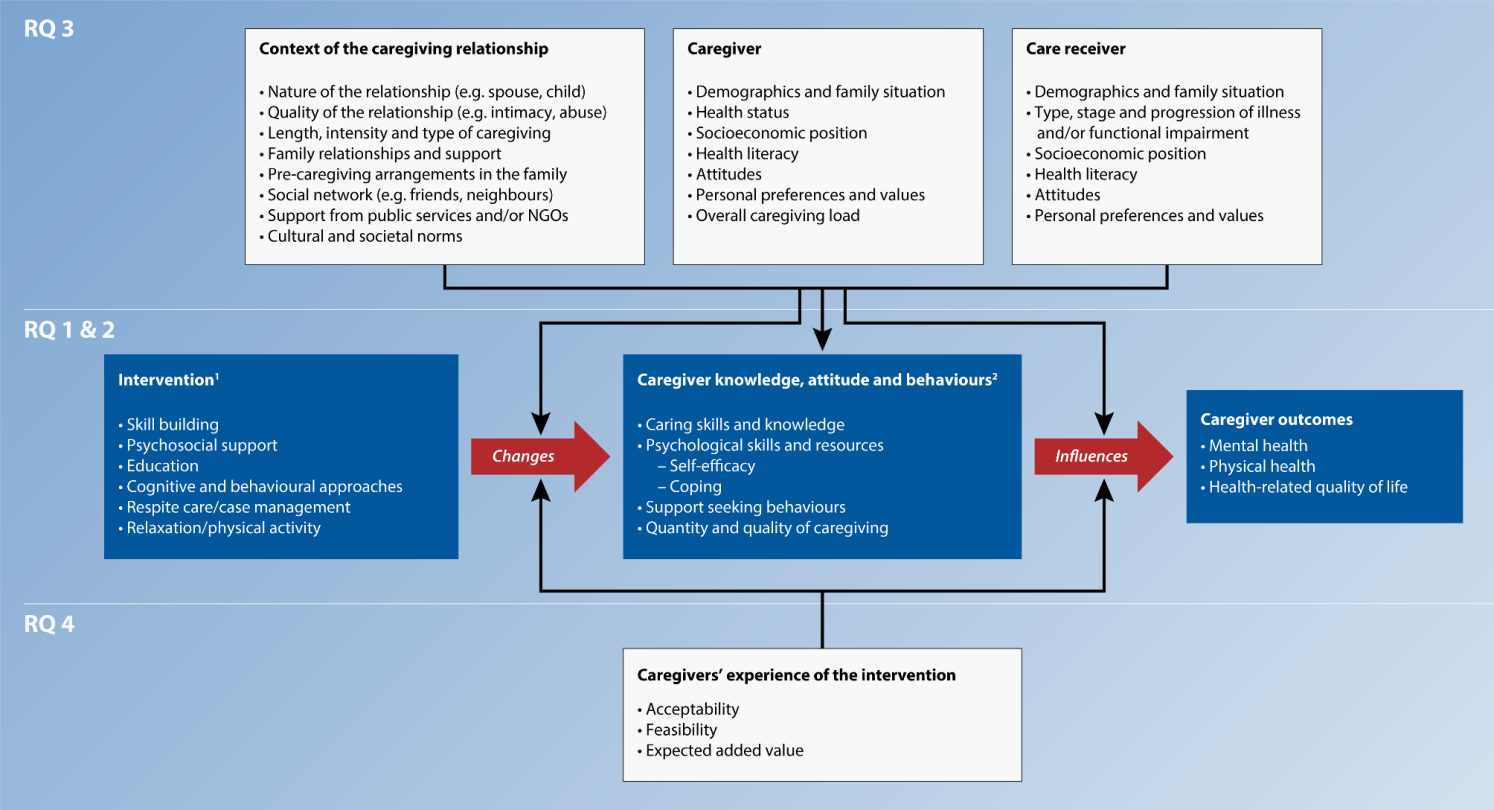
Conceptual framework linking caregiver interventions to caregiver health outcomes. The lists within the boxes may not be fully exhaustive but are rather provided to present the specific research questions driving the umbrella review. (1) Typology inspired by the work by Gaugler *et al*.^37^ (2) An adapted version of Van Houtven *et al*’s framework.^43^

An adapted version of Van Houtven *et al*’s^43^ framework will be used to organise caregiver interventions (figure 1), which will be iteratively refined from the reports of completed reviews. As a complementary output, the final framework will be displayed in the form of diagrams showing the links between interventions’ inputs and outputs.

### Patient and public involvement

No patients or members of the public were involved in the development of this umbrella review. However, the scope and methods of this review were informed by the literature and discussions with experts in the field.

### Ethics and dissemination

The proposed umbrella review will rely exclusively on published data from secondary sources and will thus not involve any interactions with human subjects. It is therefore exempt from institutional review board (IRB) approval.

The results from the umbrella review will be presented at international congresses within the fields of, for example, gerontology and geriatrics, primary care, public health and social sciences, and will be published in a journal addressing a broad readership. On publication of the results, we will make the data generated by our research openly and publicly available. The team also intends to use media services available through the participating centres and funding organisation to publicise its findings via websites, social media and newsletters.

## Discussion

The majority of care received by the European older population is provided by informal caregivers, and at least one-third of them have extensive care needs that are unmet by social services.^44^ The COVID-19 pandemic has further challenged social care for older people across the globe, unravelling its potential vulnerabilities. Informal caregivers are thus key agents in ensuring that community-living older people continue to experience a good quality of life. In the European context, this is further exacerbated by population ageing, increasing levels of dependency and cost containment.^1^

Caregiving is compatible with a low burden, but it can also exert a considerable cost on the physical and mental health of caregivers. This is especially true among older spouses caring for a highly dependent partner. Thus, if informal caregiving is to be sustainable, appropriate and timely interventions need to prevent caregivers from suffering from its negative heath consequences, on top of the risk of health decline associated with their own age.

The synthesis of the evidence proposed in this umbrella review will provide directions to strengthen the resilience of social care systems by supporting informal caregivers. An efficient coordination between viable formal and informal care will contribute to a better preparedness of the overall system of care for older people in dealing with potential future public health challenges.

## Supplementary Material

Author's manuscript
