# Supplementary material for: Effectiveness of interventions to address the negative health outcomes of informal caregiving to older adults: protocol for an umbrella review
Source: BMJ Open. 2021 Nov 9;11(11):e053117. doi: 10.1136/bmjopen-2021-053117 (PMC8578982; doi:10.1136/bmjopen-2021-053117)
Supplement: Supplementary data [file bmjopen-2021-053117supp001.pdf]

Appendix 1. Ad hoc quality appraisal checklist for qualitative reviews (adapted from Joanna Briggs Institute Checklist for Systematic Reviews and Research Syntheses[1]).

Questions	Possible responses
1. Was the research question clearly and explicitly stated?	Yes / No / Unclear / Not Applicable
2. Were the inclusion and exclusion criteria clearly described?	Yes / No / Unclear / Not Applicable
3. Was the literature search transparent and comprehensive enough to capture the relevant literature?	Yes / No / Unclear / Not Applicable
4. Did the authors use at least two databases for the literature search?	Yes / No / Unclear / Not Applicable
5. Did the authors perform a manual search of reference lists of included studies?	Yes / No / Unclear / Not Applicable
6. Was the study selection performed by two or more researchers independently?	Yes / No / Unclear / Not Applicable
7. Was the methodological quality formally assessed with explicit criteria?	Yes / No / Unclear / Not Applicable
8. Was the appraisal conducted by two or more researchers independently?	Yes / No / Unclear / Not Applicable
9. Was data extraction performed in duplicate or based on an established process of inter-author agreement assessment?	Yes / No / Unclear / Not Applicable
10. Were the synthesis methods justified?	Yes / No / Unclear / Not Applicable
11. Did the synthesis process involve two or more researchers?	Yes / No / Unclear / Not Applicable
12. Were the conclusions (including recommendations for policy and/or practice) clearly grounded in the results of the review?	Yes / No / Unclear / Not Applicable

REFERENCES

1. Aromataris E, Fernandez R, Godfrey CM, Holly C, Khalil H, Tungpunkom P. Summarizing systematic reviews: Methodological development, conduct and reporting of an umbrella review approach. *Int J Evid Based Healthc*. 2015;13(3):132-140. doi:10.1097/XEB.0000000000000055
