# Supplementary material for: Effectiveness of interventions to address the negative health outcomes of informal caregiving to older adults: protocol for an umbrella review
Source: BMJ Open. 2021 Nov 9;11(11):e053117. doi: 10.1136/bmjopen-2021-053117 (PMC8578982; doi:10.1136/bmjopen-2021-053117)
Supplement: Author's manuscript [file bmjopen-2021-053117.draft_revisions.pdf]

BMJ Open

BMJ Open is committed to open peer review. As part of this commitment we make the peer review history of every article we publish publicly available.

When an article is published we post the peer reviewers' comments and the authors' responses online. We also post the versions of the paper that were used during peer review. These are the versions that the peer review comments apply to.

The versions of the paper that follow are the versions that were submitted during the peer review process. They are not the versions of record or the final published versions. They should not be cited or distributed as the published version of this manuscript.

BMJ Open is an open access journal and the full, final, typeset and author-corrected version of record of the manuscript is available on our site with no access controls, subscription charges or pay-per-view fees (http://bmjopen.bmj.com).

If you have any questions on BMJ Open's open peer review process please email info.bmjopen@bmj.com

BMJ Open

Effectiveness of interventions to address the negative health outcomes of informal caregiving to older adults: protocol for an umbrella review

Journal:	*BMJ Open*
Manuscript ID	bmjopen-2021-053117
Article Type:	Protocol
Date Submitted by the Author:	05-May-2021
Complete List of Authors:	Calderón-Larrañaga, Amaia; Aging Research Center, Department of Neurobiology, Care Sciences and Society, Karolinska Institutet<br>Kirvalidze, Mariam; Aging Research Center, Department of Neurobiology, Care Sciences and Society, Karolinska Institutet<br>Dahlberg, Lena; Aging Research Center, Department of Neurobiology, Care Sciences and Society, Karolinska Institutet; Dalarna University, School of Education, Health and Social Studies<br>Sacco, Lawrence; Stockholm University Stress Research Institute, Department of Psychology<br>Morin, Lucas; University Hospital Centre Besancon, Inserm CIC 1431; Aging Research Center, Department of Medical Epidemiology and Biostatistics, Karolinska Institutet
Keywords:	PUBLIC HEALTH, Health policy < HEALTH SERVICES ADMINISTRATION & MANAGEMENT, QUALITATIVE RESEARCH, Clinical trials < THERAPEUTICS

SCHOLARONE™
Manuscripts

BMJ

I, the Submitting Author has the right to grant and does grant on behalf of all authors of the Work (as defined in the below author licence), an exclusive licence and/or a non-exclusive licence for contributions from authors who are: i) UK Crown employees; ii) where BMJ has agreed a CC-BY licence shall apply, and/or iii) in accordance with the terms applicable for US Federal Government officers or employees acting as part of their official duties; on a worldwide, perpetual, irrevocable, royalty-free basis to BMJ Publishing Group Ltd ("BMJ") its licensees and where the relevant Journal is co-owned by BMJ to the co-owners of the Journal, to publish the Work in this journal and any other BMJ products and to exploit all rights, as set out in our licence.

The Submitting Author accepts and understands that any supply made under these terms is made by BMJ to the Submitting Author unless you are acting as an employee on behalf of your employer or a postgraduate student of an affiliated institution which is paying any applicable article publishing charge ("APC") for Open Access articles. Where the Submitting Author wishes to make the Work available on an Open Access basis (and intends to pay the relevant APC), the terms of reuse of such Open Access shall be governed by a Creative Commons licence – details of these licences and which Creative Commons licence will apply to this Work are set out in our licence referred to above.

Other than as permitted in any relevant BMJ Author's Self Archiving Policies, I confirm this Work has not been accepted for publication elsewhere, is not being considered for publication elsewhere and does not duplicate material already published. I confirm all authors consent to publication of this Work and authorise the granting of this licence.

For peer review only

TITLE

Effectiveness of interventions to address the negative health outcomes of informal caregiving to older adults: protocol for an umbrella review

AUTHORS

Amaia Calderón-Larrañaga[1], Mariam Kirvalidze[1], Lena Dahlberg[1,2], Lawrence B. Sacco[3], Lucas Morin[4,5]

1. Aging Research Center, Department of Neurobiology, Care Sciences and Society, Karolinska Institutet, Solna, Sweden
2. School of Education, Health and Social Studies, Dalarna University, Falun, Sweden
3. Stress Research Institute, Department of Psychology, Stockholm University, Stockholm, Sweden
4. Inserm CIC 1431, University Hospital of Besançon, Besançon, France
5. Department of Medical Epidemiology and Biostatistics, Karolinska Institutet, Solna, Sweden

CORRESPONDING AUTHOR

Amaia Calderón-Larrañaga; amaia.calderon.larranaga@ki.se

Aging Research Center, Karolinska Institutet

Tomtebodavägen 18A, Floor 10, 17165 Solna, Sweden

For peer review only

ABSTRACT

Introduction

Informal caregivers play an essential role in caring for older people, whose care needs are often not fully met by healthcare and social services. While providing informal care may be a positive experience, it can also exert a considerable strain on caregivers' physical and mental health. How to best support the diverse needs of informal caregivers remains largely debated. This umbrella review aims to evaluate (1) whether effective interventions can mitigate the negative health outcomes of informal caregiving, (2) whether certain types of interventions are more effective than others, (3) whether effectiveness of interventions depends on caregiver, care receiver, context or implementation characteristics and (4) how these interventions are perceived by caregivers in terms of their acceptability, feasibility, and added value.

Methods and analysis

We will include systematic reviews—with or without meta-analysis or meta-synthesis—of primary studies focusing on the effectiveness of interventions (public or private, uni- or multi-faceted, delivered by any healthcare or social care professional or a volunteer) aimed at reducing the impact of caregiving on caregivers' physical or mental health. This includes quantitative and qualitative syntheses of implementation studies. The literature search will be undertaken using the following databases: Medline, CINAHL, PsycINFO and Web of Science. Additionally, a key informant-guided search of the grey literature will be performed. Quality appraisal will be conducted with the AMSTAR-2 checklist for quantitative reviews and with an *ad hoc* checklist for qualitative research syntheses. Narrative and tabular summaries of extracted data will be produced, and framework synthesis methods will be employed for weaving together evidence from quantitative studies in effectiveness reviews with findings on implementation and process evaluation from qualitative studies.

Ethics and dissemination

This umbrella review will use published data from secondary sources and will not involve any interactions with study participants; it is thus exempt from ethical approval. Results will be presented at international conferences and will be published in a peer-reviewed scientific journal.

PROSPERO registration number: The review protocol has been submitted for registration in PROSPERO on Monday, 3 May 2021. The registration number will be added as soon as it is available to the authors.

STRENGTHS AND LIMITATIONS OF THIS STUDY

- This umbrella review will synthesise the evidence from existing systematic reviews addressing the effectiveness of interventions to mitigate the negative health consequences of informal caregiving.
- It will include both quantitative reviews on effectiveness together with qualitative syntheses exploring complex aspects related to acceptability, feasibility and added value.
- It will account, as far as possible, for the nature and contexts of care by exploiting gender, age, ethnicity, and socioeconomic diversity perspectives and considering the characteristics of the care provided (e.g., intensity and relationship with the care receiver) across systematic reviews.
- More recent primary studies on new interventions may not be captured, as they may not yet have been synthesised into systematic reviews.

For peer review only

INTRODUCTION

Informal caregiving to older adults has become a major societal challenge globally. Aging demographics and aging-in-place-policies have led to an increased number of older people living the last part of their lives at home.[1] A considerable fraction of these individuals have complex care needs—characterised by multiple conditions, cognitive and physical impairments—and require substantial support, beyond that provided by formal care services.[2] These demands are especially intense in times of public health crisis, such as the COVID-19 pandemic during which many older people have been asked to self-isolate in their homes.[3]

The number of informal (unpaid) caregivers has grown fast over the last years, contributing to the majority of the care received by those aged over 50 in most European countries,[4] and it is expected that the number of people taking up the caregiver role will increase in the next years.[5] Despite the noticeable increase in the use of privately purchased care services, these mostly play a marginal role.[6] Instead, a major trend towards the *informalisation* of social care has been observed since the 1990s.[7,8] While providing informal care may be lived as a positive experience,[9] it can also be distressing and have negative health consequences.[10] A state of subjective burden is characterised by fatigue and stress in the caregiver,[11] and by a higher risk of institutionalisation and psychiatric symptoms in the care receiver.[12]

The literature regarding the management of caregiver burden is evolving rapidly and an increasing number of primary studies and systematic reviews assessing the effectiveness of different interventions have been published.[1] Yet, we still do not know how best to support the diverse needs of informal caregivers throughout the course of their 'invisible journey' in caregiving.[13,14] This is mainly due to a lack of nuance in understanding how their needs may differ depending on their experience of caregiving. Several factors play an essential role in shaping this intimate and highly personal experience. The development of the *stress process*[15] among caregivers is influenced by the intensity of care demands, the length and current stage of the caregiving journey, the existence of other sources of strain, the caregivers' coping style and the density of their social network.[16] The consequences of informal caregiving also vary depending on the relationship between the care receiver and the caregiver, and on the acute or chronic nature of the care receiver's condition.[17]

Therefore, in designing caregiver interventions, it is essential to distinguish between elements that might be broadly applicable to all informal caregivers at large and elements that are specific to certain caregivers, care receivers' health problems and contexts of care.[18] Accordingly, a systematic review of 31 randomised controlled trials in the context of dementia and Alzheimer's disease found insufficient evidence to endorse the use of most interventions but noted larger trials that employed tailored interventions had significant effects on at least one outcome.[19] This exemplifies the limitation of taking a 'blanket approach' to designing interventions for caregivers at large, as these might fail to recognise significant effects on subsets of the caregiver population, thereby underestimating the wide heterogeneity in informal care provision and in the circumstances of caregivers.

Systematic reviews and meta-analyses of caregiving interventions often consider study populations as homogeneous, which masks contextual factors such as culture, financial resources, or health literacy that impact caregivers' risk of negative physical and mental outcomes.[20] This one-size-fits-all approach has been shown to be inadequate, but remains common in informal caregiving research.[21] We believe an umbrella review (i.e. a review of systematic reviews) is a necessary step that will provide a broader picture of the effectiveness of different types of support services targeting different pools of informal caregivers. This requires combining an "aggregative approach", whereby quantitative reviews are used to answer well-defined questions based on empirical observations, together with more "configurative approaches" drawing not only on quantitative but also qualitative reviews.[22]

The aim of this umbrella review is to synthesise the evidence from existing systematic reviews evaluating the effectiveness of interventions to address the negative health consequences of informal caregiving. In particular, the following research questions will be addressed: 1) Are there effective interventions to prevent and reduce the negative health consequences of informal caregiving?; 2) Are certain types of interventions more effective than others?; 3) Is there evidence that the effectiveness of interventions depends on

For peer review only - http://bmjopen.bmj.com/site/about/guidelines.xhtml

caregiver, care receiver, care context and implementation characteristics?; and 4) How are the proposed interventions experienced by caregivers in terms of their acceptability, feasibility and added value?

METHODS AND ANALYSIS

Study design

We will conduct an umbrella review, defined as "the compilation of evidence from pre-existing systematic reviews into one accessible and usable document".[23] Umbrella reviews focus on broad conditions or problems (e.g. interventions to mitigate the negative health consequences of informal caregiving) for which there is a rich and high-quality evidence base. The bird's-eye view provided by an umbrella review is well-suited to examine whether the evidence base around a given research question is consistent or contradictory, and to explore the underlying reasons in detail.[24] Furthermore, it will enable us to assess whether review authors who worked independently from each other but addressed similar research questions observed similar results and drew similar conclusions. This umbrella review will be reported in accordance with the Preferred Reporting Items for Overviews of Review (PRIOR) statement.[25]

Target population

We will include systematic reviews of both quantitative and qualitative primary studies with or without meta-analysis focusing on interventions to address the negative health consequences of informal caregiving for informal caregivers of older adults. The full list of inclusion and exclusion criteria developed for this umbrella review is provided in **Table 1**.

For the purpose of this umbrella review, informal caregiver will be defined as any relative, partner, friend or neighbour who has a significant personal relationship with, and provides a broad range of assistance for, an older person with a chronic or disabling condition.[26] Some have argued that the term 'family caregiver' should be preferred over 'informal caregiver' on the ground that caregivers may find the latter invalidating and belittling.[27] Although we acknowledge the importance of terminology around this complex issue, this umbrella review will use the term 'informal caregiving' to explicitly include non-familial caregivers in its scope. Older care receivers will be defined by chronological age or as having an age-related disease or syndrome such as dementia, cancer, stroke, Parkinson's disease, heart failure, multimorbidity and/or frailty. This corresponds with the broad range of caregiver experiences and needs that healthcare and social care providers tend to encounter. We will exclude reviews focusing exclusively on interventions directed towards care receivers. Reviews of interventions specific to the context of end-of-life care will also be excluded, since caregiving approaches at this stage of the illness trajectory warrant a separate review and discussion.[28]

Interventions, comparators and outcomes

Systematic reviews will be included if they provide synthesised results about the effectiveness, implementation or process of interventions aimed at preventing and reducing negative health outcomes in the caregivers. We will examine all types of interventions (public or private), whether they are uni- or multi-faceted interventions, and delivered by all types of healthcare or social care professionals or volunteers. The typology developed by Gaugler et al[29] will be adapted to categorise intervention types: skill building, psychosocial support, education, cognitive and behavioural approaches, respite, care/case management, and relaxation/physical activity.

In those reviews where a comparator is required, any comparator(s) tested will be considered: intervention versus control (no intervention, usual care, placebo or other control, or another intervention) as defined by the original reviews. We will include different caregiver-related physical and mental health outcomes, since we want to give an overview of the different outcome measures used in the included reviews. We will exclude reviews measuring exclusively non-health related outcomes, such as caregiver burden, family relations, work and financial status or stress/strain in the caregiver.

Publication type, date and language

For peer review only - http://bmjopen.bmj.com/site/about/guidelines.xhtml

Reviews published in a peer-reviewed journal or as a report done or mandated by an official health agency (e.g. Health Technology Assessment agency) will be included. Even if the first reviews on caregiving intervention research were published in the 1990s, we will focus on reviews published since 2000 in an attempt to capture studies taking place in the context of recent social changes and advancements in caregiving intervention research. We will restrict the inclusion to reviews published in languages spoken by research team members: English, Swedish, Spanish, French, Italian and German.

Data sources and search strategy

An iterative search strategy for bibliometric databases and grey literature will be developed to look for peer-reviewed systematic reviews evaluating interventions addressing the negative health consequences in the informal caregiver in all care settings, including quantitative and/or qualitative syntheses of implementation studies and process evaluations alongside trials testing such interventions. Therefore, the search for this umbrella review will aim to identify all research syntheses relevant to our four research questions. The search strategy will be guided by a librarian, comprehensively reported and the detailed search filters employed will be presented sequentially in the single appendix for all the databases that were searched and listed along with the search dates. The literature search will be undertaken using the following biomedical and sociological citation databases: Medline, CINAHL, PsycINFO and Web of Science. We will use *CoCites* (https://www.cocites.com/) citation-based search tool to widen the net and retrieve articles that cite eligible systematic reviews.[30]

The preliminary search strategy is detailed in In **Table 2**. This search strategy has been developed by the research team with the guidance of experienced health sciences librarians to estimate the number of potentially relevant systematic reviews. Additional searches will be developed for syntheses of effectiveness, implementation or process evaluations published or mandated by official health agencies. We also expect a substantial number of such reports meeting our criteria not to be necessarily published in peer-reviewed journals. Thus, we will contact the first and last authors of selected reviews to retrieve grey literature that may otherwise have been missed. Finally, we will perform a manual search of the reference lists of relevant reviews.

Data collection

Titles and abstracts of the citations will be screened by two independent reviewers by using the Covidence (https://www.covidence.org/) software developed by the Cochrane collaboration.[31] Before screening, inclusion criteria will be tested in a pilot study. We will report any changes to the inclusion and exclusion criteria that result from the calibration exercise as deviations from the published protocol. Full texts of the relevant documents will be independently checked for eligibility by two independent reviewers. Any dissent in abstract screening and full-text assessment will be resolved by discussion moderated by a third reviewer. Reviews excluded during the full-text assessment will be documented along with the reason for exclusion.

Methodological quality and risk of bias assessment

Critical appraisal of quantitative reviews will be conducted independently by two reviewers using the AMSTAR-2 checklist.[32] The checklist categorises the quality of the reviews based on seven critical and nine non-critical domains.[32] Based on the appraisal, the reviews will be grouped into critically low, low, moderate and high quality categories. Qualitative reviews will be assessed by two reviewers using an *ad hoc* quality appraisal checklist (**Appendix 1**), adapted from the Joanna Briggs Institute Checklist for Systematic Reviews and Research Syntheses.[24] The tool has been developed and piloted by all members of our multidisciplinary group. Any dissent in the process of quality assessment will be resolved through discussions moderated by a third reviewer.

Data extraction

Data will be extracted independently by two reviewers according to the type of systematic review (i.e. systematic reviews of qualitative studies, mixed methods studies, and/or quantitative studies). Data extraction will be calibrated during a pilot phase, with a purposive sample of five studies, to ensure that all relevant data are extracted in a consistent manner. Extraction will be limited to data reported in the included

For peer review only - http://bmjopen.bmj.com/site/about/guidelines.xhtml

reviews. Clear indication of overlap of primary research studies in each of the included reviews will be presented, and significant overlap will be addressed to avoid double counting.

Data synthesis

A narrative description of the included reviews will be provided, with reference to a detailed table of included review characteristics. This description will allow for contextualizing the results in terms of the relevance and evidence base of included research syntheses for the umbrella review questions. Specific items or points of interest from individual reviews will also be highlighted, as described below.

Overall effect estimates from quantitative systematic reviews will be presented in tables, alongside a detailed description of the interventions assessed. In addition to the direction and magnitude of effects, number of reviews that inform each outcome, number of participants, and statistical heterogeneity, factors related to the caregiver, care receiver and/or care context affecting the effectiveness of interventions will be identified whenever possible.

Evidence from syntheses of qualitative research will also be presented in tabular format. Results will be synthesised and illustrated by using verbatim replications from the source review when appropriate. Findings will also be described and interpreted in the research team's own words. Caregivers' experiences and views on barriers to and facilitators of interventions will be identified in terms of acceptability, feasibility and added value. The diversity of caregivers, care receivers and/or care contexts will be considered as far as possible.

Framework synthesis methods will be employed for the integration of quantitative studies in effectiveness reviews with qualitative and process evaluation evidence. These methods are particularly suitable for capturing complexity of interventions in systematic reviews.[33]

An adapted version of Van Houtven et al's[34] framework will be used to organize caregiver interventions (**Figure 1**), which will be iteratively refined from the reports of completed reviews. As a complementary output, the final framework will be displayed in the form of diagrams showing the links between interventions' inputs and outputs.

Patient and public involvement (PPI)

No patients or members of the public were involved in the development of this umbrella review. However, the scope and methods of this review were informed by the literature and discussions with experts in the field.

Ethics and dissemination

The proposed umbrella review will rely exclusively on published data from secondary sources and will thus not involve any interactions with human subjects. It is therefore exempt from institutional review board (IRB) approval.

The results from the umbrella review will be presented at international congresses within the fields of e.g. gerontology and geriatrics, primary care, public health and social sciences, and will be published in a journal addressing a broad readership. Upon publication of the results, we will make the data generated by our research openly and publicly available. The team also intends to use media services available through the participating centres and funding organisation to publicise its findings via websites, social media and newsletters.

DISCUSSION

The majority of care received by the European older population is provided by informal family caregivers, and at least one third of them have extensive care needs that are unmet by social services.[35] The COVID-19 pandemic has further challenged social care for older people across the globe, unravelling its potential vulnerabilities. Informal caregivers are thus key agents in ensuring that community-living older people continue to experience a good quality of life. In the European context, this is further exacerbated by population aging, increasing levels of dependency, and cost containment.[1]

For peer review only - http://bmjopen.bmj.com/site/about/guidelines.xhtml

Caregiving is compatible with a low burden, but it can also exert a considerable cost on the physical and mental health of caregivers. This is especially true among older spouses caring for a highly dependent partner. Thus, if informal caregiving is to be sustainable, appropriate and timely, interventions need to prevent caregivers from suffering from its negative heath consequences, on top of the risk of health decline associated with their own age.

The synthesis of the evidence proposed in this umbrella review will provide directions to strengthen the resilience of social care system by supporting informal caregivers. An efficient coordination between viable formal and informal care will contribute to a better preparedness of the overall system of care for older people in dealing with potential future public health challenges.

For peer review only

AUTHORS' CONTRIBUTIONS: AC-L drafted the protocol. All authors participated in conceptualising the methodology, provided critical revisions and approved the final version of the protocol for submission.

ACKNOWLEDGMENTS: We wish to thank Emma-Lotta Säätelä and Jonas Pettersson, librarians at Karolinska Institutet, for preparing the search strategy and performing the searches across databases.

FUNDING STATEMENT: This work was supported by the Swedish Research Council for Health, Working Life and Welfare (FORTE) grant number 2020-01544.

COMPETING INTERESTS STATEMENT: None declared.

WORD COUNT: 2644

For peer review only

REFERENCES

1. Colombo F, Llena-Nozal A, Mercier J, Tjadens F. *Help Wanted?: Providing and Paying for Long-Term Care, OECD Health Policy Studies*. OECD Publishing; 2011.
2. Pavolini E, Ranci C. Restructuring the welfare state: reforms in long-term care in Western European countries. *J Eur Soc Policy*. 2008;18(3):246-259. doi:10.1177/0958928708091058
3. Chan EYY, Gobat N, Kim JH, et al. Informal home care providers: the forgotten health-care workers during the COVID-19 pandemic. *Lancet*. 2020;395(10242):1957-1959. doi:10.1016/S0140-6736(20)31254-X
4. Verbeek-Oudijk D, Woittiez I, Eggink E, Putman L. *Who Cares in Europe? A Comparison of Long-Term Care for the over-50 s in Sixteen European Countries*.; 2014.
5. Broese van Groenou MI, De Boer A. Providing informal care in a changing society. *Eur J Ageing*. 2016;13(3):271-279. doi:10.1007/s10433-016-0370-7
6. Le Bihan B, Da Roit B, Sopadzhiyan A. The turn to optional familialism through the market: Long-term care, cash-for-care, and caregiving policies in Europe. *Soc Policy Adm*. 2019;53(4):579-595. doi:10.1111/spol.12505
7. Le Bihan B, Martin C. Diversification of care policy measures supporting older people: towards greater flexibility for carers? *Eur J Ageing*. 2012;9(2):141-150. doi:10.1007/s10433-012-0228-6
8. Szebehely M, Trydegård G-B. Home care for older people in Sweden: a universal model in transition. *Health Soc Care Community*. 2012;20(3):300-309. doi:10.1111/j.1365-2524.2011.01046.x
9. Roth DL, Fredman L, Haley WE. Informal Caregiving and Its Impact on Health: A Reappraisal From Population-Based Studies. *Gerontologist*. 2015;55(2):309-319. doi:10.1093/geront/gnu177
10. Hiel L, Beenackers MA, Renders CM, Robroek SJW, Burdorf A, Croezen S. Providing personal informal care to older European adults: Should we care about the caregivers' health? *Prev Med (Baltim)*. 2015;70:64-68. doi:10.1016/j.ypmed.2014.10.028
11. del-Pino-Casado R, Rodríguez Cardosa M, López-Martínez C, Orgeta V. The association between subjective caregiver burden and depressive symptoms in carers of older relatives: A systematic review and meta-analysis. Cheungpasitporn W, ed. *PLoS One*. 2019;14(5):e0217648. doi:10.1371/journal.pone.0217648
12. Martín-Carrasco M, Martín MF, Valero CP, et al. Effectiveness of a psychoeducational intervention program in the reduction of caregiver burden in alzheimer's disease patients' caregivers. *Int J Geriatr Psychiatry*. 2009;24(5):489-499. doi:10.1002/gps.2142
13. Schulz R, Eden J, eds. *Families Caring for an Aging America*. National Academies Press; 2016.
14. Williams F, Moghaddam N, Ramsden S, De Boos D. Interventions for reducing levels of burden amongst informal carers of persons with dementia in the community. A systematic review and meta-analysis of randomised controlled trials. *Aging Ment Health*. 2019;23(12):1629-1642. doi:10.1080/13607863.2018.1515886
15. Pearlin LI, Mullan JT, Semple SJ, Skaff MM. Caregiving and the Stress Process: An Overview of Concepts and Their Measures. *Gerontologist*. 1990;30(5):583-594. doi:10.1093/geront/30.5.583
16. Mello J de A, Macq J, Van Durme T, et al. The determinants of informal caregivers' burden in the care of frail older persons: a dynamic and role-related perspective. *Aging Ment Health*. 2017;21(8):838-843. doi:10.1080/13607863.2016.1168360
17. Conde-Sala JL, Garre-Olmo J, Turró-Garriga O, Vilalta-Franch J, López-Pousa S. Differential features of burden between spouse and adult-child caregivers of patients with Alzheimer's disease: An exploratory comparative design. *Int J Nurs Stud*. 2010;47(10):1262-1273. doi:10.1016/j.ijnurstu.2010.03.001
18. Young HM, Bell JF, Whitney RL, Ridberg RA, Reed SC, Vitaliano PP. Social Determinants of Health: Underreported Heterogeneity in Systematic Reviews of Caregiver Interventions. Hepburn K, ed. *Gerontologist*. 2020;60(Supplement_1):S14-S28. doi:10.1093/geront/gnz148
19. Griffin J, Meis L, Greer N, et al. *Effectiveness of Family and Caregiver Interventions on Patient Outcomes Among Adults with Cancer or Memory-Related Disorders: A Systematic Review*.; 2013.
20. Bom J, Bakx P, Schut F, van Doorslaer E. The Impact of Informal Caregiving for Older Adults on the Health of Various Types of Caregivers: A Systematic Review. *Gerontologist*. Published online November 2018. doi:10.1093/geront/gny137
21. Hepburn K, Siegel EO. New Directions to Advance Family Caregiving Research. *Gerontologist*. 2020;60(Supplement_1):S1-S4. doi:10.1093/geront/gnz182
22. Petticrew M, Rehfuess E, Noyes J, et al. Synthesizing evidence on complex interventions: how meta-analytical, qualitative, and mixed-method approaches can contribute. *J Clin Epidemiol*. 2013;66(11):1230-1243. doi:10.1016/j.jclinepi.2013.06.005
23. Grant MJ, Booth A. A typology of reviews: an analysis of 14 review types and associated

For peer review only

For peer review only - http://bmjopen.bmj.com/site/about/guidelines.xhtml

methodologies. *Heal Inf Libr J*. 2009;26(2):91-108. doi:10.1111/j.1471-1842.2009.00848.x
24. Aromataris E, Fernandez R, Godfrey CM, Holly C, Khalil H, Tungpunkom P. Summarizing systematic reviews. *Int J Evid Based Healthc*. 2015;13(3):132-140. doi:10.1097/XEB.0000000000000055
25. Pollock M, Fernandes RM, Pieper D, et al. Preferred Reporting Items for Overviews of Reviews (PRIOR): a protocol for development of a reporting guideline for overviews of reviews of healthcare interventions. *Syst Rev*. 2019;8(1):335. doi:10.1186/s13643-019-1252-9
26. U.S. Family Caregiver Alliance. Definitions.
27. Stall NM, Campbell A, Reddy M, Rochon PA. Words Matter: The Language of Family Caregiving. *J Am Geriatr Soc*. 2019;67(10):2008-2010. doi:10.1111/jgs.15988
28. Penrod J, Hupcey JE, Baney BL, Loeb SJ. End-of-life caregiving trajectories. *Clin Nurs Res*. 2011;20(1):7-24. doi:10.1177/1054773810384852
29. Gaugler JE, Jutkowitz E, Shippee TP, Brasure M. Consistency of dementia caregiver intervention classification: an evidence-based synthesis. *Int Psychogeriatrics*. 2017;29(1):19-30. doi:10.1017/S1041610216001514
30. Janssens ACJW, Gwinn M, Brockman JE, Powell K, Goodman M. Novel citation-based search method for scientific literature: a validation study. *BMC Med Res Methodol*. 2020;20(1):25. doi:10.1186/s12874-020-0907-5
31. Veritas Health Innovation. Covidence systematic review software. Published online 2021.
32. Shea BJ, Reeves BC, Wells G, et al. AMSTAR 2: a critical appraisal tool for systematic reviews that include randomised or non-randomised studies of healthcare interventions, or both. *BMJ*. Published online September 2017:j4008. doi:10.1136/bmj.j4008
33. Harden A, Thomas J, Cargo M, et al. Cochrane Qualitative and Implementation Methods Group guidance series—paper 5: methods for integrating qualitative and implementation evidence within intervention effectiveness reviews. *J Clin Epidemiol*. 2018;97:70-78. doi:10.1016/j.jclinepi.2017.11.029
34. Van Houtven CH, Voils CI, Weinberger M. An organizing framework for informal caregiver interventions: detailing caregiving activities and caregiver and care recipient outcomes to optimize evaluation efforts. *BMC Geriatr*. 2011;11(1):77. doi:10.1186/1471-2318-11-77
35. Bien B, McKee KJ, Dohner H, et al. Disabled older people's use of health and social care services and their unmet care needs in six European countries. *Eur J Public Health*. 2013;23(6):1032-1038. doi:10.1093/eurpub/cks190

For peer review only

For peer review only - http://bmjopen.bmj.com/site/about/guidelines.xhtml

FIGURES

Figure 1. Conceptual framework linking caregiver interventions to caregiver health outcomes.

The lists within the boxes may not be fully exhaustive but are rather provided to present the specific research question driving the umbrella review.
[1]Typology inspired by the work by Gaugler et al.[29]
[2]An adapted version of Van Houtven et al's framework.[34]

For peer review only

Table 1. Inclusion and exclusion criteria for quantitative and qualitative reviews.

**Inclusion criteria**
Publication type, date and language
• Reviews published in a peer-reviewed journal OR as a report done or mandated by an official health agency (*e.g. Health Technology Assessment agency*)<br>• Reviews published between January 1, 2000 and December 31, 2020<br>• Reviews published in English, Swedish, Spanish, French, Italian or German
Study design
• For quantitative reviews: reviews including a reproducible, systematic search strategy, AND clearly defined inclusion/exclusion criteria AND risk of bias assessment for all included primary studies<br>• For qualitative reviews: reviews including a reproducible, systematic search strategy, AND defined inclusion/exclusion criteria
Population
• Reviews concerning informal caregivers (*i.e. people who regularly provide unpaid care to a family member, friend or neighbour*) of older people OR of persons presenting with aging-related disease (*e.g. dementia, stroke, Parkinson's disease, cancer, heart failure, multimorbidity, frailty*)
Intervention
• Reviews focusing on interventions and assessing either their effectiveness (for quantitative reviews) or their implementation and/or the lived experience of the target population (for qualitative reviews)
Outcome
• Reviews including physical or mental health-related outcomes of informal caregivers, including health-related quality of life
**Exclusion criteria**
• Reviews of interventions focusing *exclusively* on care receivers as the target population<br>• Reviews focusing *exclusively* on interventions for caregivers of young populations<br>• Reviews measuring *exclusively* non-health related outcomes, such as caregiver burden, stress/strain, work or financial status, family relations, breakdown of informal care<br>• Reviews focusing exclusively on end-of-life care interventions

For peer review only

Table 2. Search strategy developed by the research team to estimate the sample size. Database(s): Ovid MEDLINE(R) and Epub Ahead of Print, In-Process, In-Data-Review & Other Non-Indexed Citations and Ovid MEDLINE(R) Daily 1946 to March 26, 2021.

#	Searches
1	exp Aged/
2	exp Aging/
3	Frailty/
4	(advanced age or aged or ageing or aging or elder* or frail* or geriatr* or gerontolog* or late* life or old age or old* adult* or old* client* or old* individual* or old* man or old* men or old* patient* or old* people or old* person* or old* population* or old* woman or old* women or oldest old or retired or senior*).ti,ab,kf.
5	Dementia/
6	Alzheimer Disease/
7	(dementia* or alzheimer*).ti,ab,kf.
8	exp Neoplasms/
9	(neoplasm* or cancer*).ti,ab,kf.
10	exp Stroke/
11	stroke.ti,ab,kf.
12	Parkinson disease/
13	parkinson*.ti,ab,kf.
14	Multimorbidity/
15	multimorbid*.ti,ab,kf.
16	exp Heart failure/
17	(heart failure or cardiac failure).ti,ab,kf.
18	or/1-17
19	Caregivers/
20	(caregiv* or care giv* or caretak* or care tak* or carer*).ti,ab,kf.
21	((family or informal or unpaid) adj3 (care or caring)).ti,ab,kf.
22	19 or 20 or 21
23	(meta analysis or systematic review).pt.
24	review.ti.
25	systematic* review*.ab,kf.
26	(meta analy* or metaanaly* or meta stud* or meta interpretation* or meta ethnograph* or meta summar* or meta synthes* or meta narrative* or mixed research synthes*).ti,ab,kf.
27	((concept analy* or grounded theory) and review*).ti,ab,kf.
28	or/23-27
29	18 and 22 and 28
30	(english or swedish or spanish or french or italian or german).lg.
31	29 and 30
32	limit 31 to yr="2000 -Current"
33	limit 32 to (comment or congress or editorial or letter)
34	32 not 33

Field labels: exp/ = exploded MeSH term; / = non exploded MeSH term; .ti,ab,kf. = title, abstract and author keywords; .pt. = publication type; adjx = within x words, regardless of order; * = truncation of word for alternate endings

For peer review only

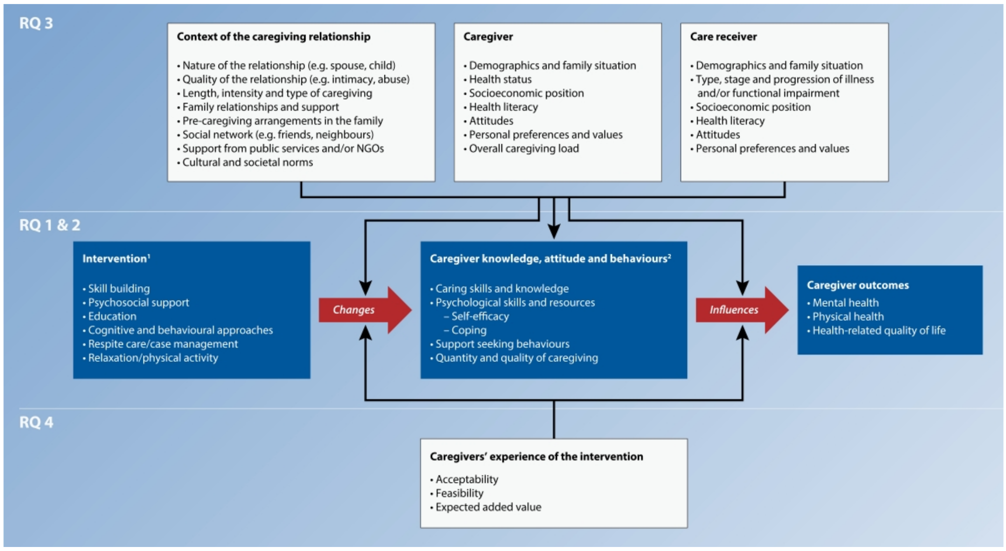

Figure 1. Conceptual framework linking caregiver interventions to caregiver health outcomes. The lists within the boxes may not be fully exhaustive but are rather provided to present the specific research question driving the umbrella review.

[1]Typology inspired by the work by Gaugler et al.[29]

[2]An adapted version of Van Houtven et al's framework.[34]

Appendix 1. Ad hoc quality appraisal checklist for qualitative reviews (adapted from Joanna Briggs Institute Checklist for Systematic Reviews and Research Syntheses[1]).

Questions	Possible responses
1. Was the research question clearly and explicitly stated?	Yes / No / Unclear / Not Applicable
2. Were the inclusion and exclusion criteria clearly described?	Yes / No / Unclear / Not Applicable
3. Was the literature search transparent and comprehensive enough to capture the relevant literature?	Yes / No / Unclear / Not Applicable
4. Did the authors use at least two databases for the literature search?	Yes / No / Unclear / Not Applicable
5. Did the authors perform a manual search of reference lists of included studies?	Yes / No / Unclear / Not Applicable
6. Was the study selection performed by two or more researchers independently?	Yes / No / Unclear / Not Applicable
7. Was the methodological quality formally assessed with explicit criteria?	Yes / No / Unclear / Not Applicable
8. Was the appraisal conducted by two or more researchers independently?	Yes / No / Unclear / Not Applicable
9. Was data extraction performed in duplicate or based on an established process of inter-author agreement assessment?	Yes / No / Unclear / Not Applicable
10. Were the synthesis methods justified?	Yes / No / Unclear / Not Applicable
11. Did the synthesis process involve two or more researchers?	Yes / No / Unclear / Not Applicable
12. Were the conclusions (including recommendations for policy and/or practice) clearly grounded in the results of the review?	Yes / No / Unclear / Not Applicable

For peer review only

REFERENCES

1. Aromataris E, Fernandez R, Godfrey CM, Holly C, Khalil H, Tungpunkom P. Summarizing systematic reviews: Methodological development, conduct and reporting of an umbrella review approach. *Int J Evid Based Healthc*. 2015;13(3):132-140. doi:10.1097/XEB.0000000000000055

For peer review only

BMJ Open

Effectiveness of interventions to address the negative health outcomes of informal caregiving to older adults: protocol for an umbrella review

Journal:	*BMJ Open*
Manuscript ID	bmjopen-2021-053117.R1
Article Type:	Protocol
Date Submitted by the Author:	13-Sep-2021
Complete List of Authors:	Calderón-Larrañaga, Amaia; Aging Research Center, Department of Neurobiology, Care Sciences and Society, Karolinska Institutet<br>Kirvalidze, Mariam; Aging Research Center, Department of Neurobiology, Care Sciences and Society, Karolinska Institutet<br>Dahlberg, Lena; Aging Research Center, Department of Neurobiology, Care Sciences and Society, Karolinska Institutet; Dalarna University, School of Health and Welfare<br>Sacco, Lawrence; Stockholm University Stress Research Institute, Department of Psychology<br>Morin, Lucas; University Hospital Centre Besancon, Inserm CIC 1431; Aging Research Center, Department of Medical Epidemiology and Biostatistics, Karolinska Institutet
**Primary Subject Heading**:	Public health
Secondary Subject Heading:	Public health, Geriatric medicine
Keywords:	PUBLIC HEALTH, Health policy < HEALTH SERVICES ADMINISTRATION & MANAGEMENT, QUALITATIVE RESEARCH, Clinical trials < THERAPEUTICS

SCHOLARONE™ Manuscripts

I, the Submitting Author has the right to grant and does grant on behalf of all authors of the Work (as defined in the below author licence), an exclusive licence and/or a non-exclusive licence for contributions from authors who are: i) UK Crown employees; ii) where BMJ has agreed a CC-BY licence shall apply, and/or iii) in accordance with the terms applicable for US Federal Government officers or employees acting as part of their official duties; on a worldwide, perpetual, irrevocable, royalty-free basis to BMJ Publishing Group Ltd ("BMJ") its licensees and where the relevant Journal is co-owned by BMJ to the co-owners of the Journal, to publish the Work in this journal and any other BMJ products and to exploit all rights, as set out in our licence.

The Submitting Author accepts and understands that any supply made under these terms is made by BMJ to the Submitting Author unless you are acting as an employee on behalf of your employer or a postgraduate student of an affiliated institution which is paying any applicable article publishing charge ("APC") for Open Access articles. Where the Submitting Author wishes to make the Work available on an Open Access basis (and intends to pay the relevant APC), the terms of reuse of such Open Access shall be governed by a Creative Commons licence – details of these licences and which Creative Commons licence will apply to this Work are set out in our licence referred to above.

Other than as permitted in any relevant BMJ Author's Self Archiving Policies, I confirm this Work has not been accepted for publication elsewhere, is not being considered for publication elsewhere and does not duplicate material already published. I confirm all authors consent to publication of this Work and authorise the granting of this licence.

For peer review only

TITLE

Effectiveness of interventions to address the negative health outcomes of informal caregiving to older adults: protocol for an umbrella review

AUTHORS

Amaia Calderón-Larrañaga[1], Mariam Kirvalidze[1], Lena Dahlberg[1,2], Lawrence B. Sacco[3], Lucas Morin[4,5]

1. Aging Research Center, Department of Neurobiology, Care Sciences and Society, Karolinska Institutet, Solna, Sweden
2. School of Health and Welfare, Dalarna University, Falun, Sweden
3. Stress Research Institute, Department of Psychology, Stockholm University, Stockholm, Sweden
4. Inserm CIC 1431, University Hospital of Besançon, Besançon, France
5. Department of Medical Epidemiology and Biostatistics, Karolinska Institutet, Solna, Sweden

CORRESPONDING AUTHOR

Amaia Calderón-Larrañaga; amaia.calderon.larranaga@ki.se

Aging Research Center, Karolinska Institutet

Tomtebodavägen 18A, Floor 10, 17165 Solna, Sweden

For peer review only

ABSTRACT

Introduction

Informal (unpaid) caregivers play an essential role in caring for older people, whose care needs are often not fully met by formal services. While providing informal care may be a positive experience, it can also exert a considerable strain on caregivers' physical and mental health. How to best support the needs of informal caregivers remains largely debated. This umbrella review (review of systematic reviews) aims to evaluate (1) whether effective interventions can mitigate the negative health outcomes of informal caregiving, (2) whether certain types of interventions are more effective than others, (3) whether effectiveness of interventions depends on caregiver/receiver, context, or implementation characteristics and (4) how these interventions are perceived in terms of acceptability, feasibility, and added value.

Methods and analysis

We will include systematic reviews of primary studies focusing on the effectiveness of interventions (public or private, uni- or multi-faceted, delivered by health or social care professionals or volunteers) aimed at reducing the impact of caregiving on caregivers' physical or mental health. This will also include quantitative and qualitative syntheses of implementation studies. The literature search will include the following databases: Medline, CINAHL, PsycINFO and Web of Science. A key informant-guided search of grey literature will be performed. Quality appraisal will be conducted with the AMSTAR-2 checklist for quantitative reviews and with an *ad hoc* checklist for qualitative syntheses. Narrative and tabular summaries of extracted data will be produced, and framework synthesis will be employed for weaving together evidence from quantitative studies in effectiveness reviews with findings on implementation from qualitative studies.

Ethics and dissemination

This umbrella review will use data from secondary sources and will not involve interactions with study participants; it is thus exempt from ethical approval. Results will be presented at international conferences and will be published in a peer-reviewed journal.

Registration

CRD42021252841

STRENGTHS AND LIMITATIONS OF THIS STUDY

- The umbrella review methodology will enable us to perform higher level analysis by synthesising the evidence from existing systematic reviews addressing the effectiveness of interventions to mitigate the negative health consequences of informal caregiving.
- It will be possible to include both quantitative reviews on effectiveness together with qualitative syntheses exploring complex aspects related to acceptability, feasibility and added value.
- Due to heterogeneity in review designs, findings will not be directly comparable, and synthesis might be confined to a narrative output.
- More recent primary studies on new interventions may not be captured, as they may not yet have been included in systematic reviews.

For peer review only

For peer review only - http://bmjopen.bmj.com/site/about/guidelines.xhtml

INTRODUCTION

Informal caregiving to older adults has become a major societal challenge globally. Aging demographics and aging-in-place-policies have led to an increased number of older people living the last part of their lives at home.[1] A considerable fraction of these individuals have complex care needs—characterised by multiple conditions, cognitive and physical impairments—and require substantial support, beyond that provided by formal care services.[2] These demands are especially intense in times of public health crisis, such as the COVID-19 pandemic during which many older people have been asked to self-isolate in their homes.[3]

The number of informal (unpaid) caregivers has grown fast over the last years, contributing to the majority of the care received by those aged over 50 in most European countries,[4] and it is expected that the number of people taking up the caregiver role will increase in the next years.[5] Despite the noticeable increase in the use of privately purchased care services, these mostly play a marginal role.[6] Instead, a major trend towards the *informalisation* of social care has been observed since the 1990s.[7,8] While providing informal care may be lived as a positive experience,[9] it can also be distressing and have negative health consequences.[10] A state of subjective burden is characterised by fatigue and stress in the caregiver,[11] and by a higher risk of institutionalisation and psychiatric symptoms in the care receiver.[12]

The literature regarding the management of caregiver burden is evolving rapidly and an increasing number of primary studies and systematic reviews assessing the effectiveness of different interventions have been published.[13–15] Yet, we still do not know how best to support the diverse needs of informal caregivers throughout the course of their 'invisible journey' in caregiving.[16,17] This is mainly due to a lack of nuance in understanding how their needs may differ depending on their experience of caregiving. Several factors play an essential role in shaping this intimate and highly personal experience. The development of the *stress process*[18] among caregivers is influenced by the intensity of care demands, the length and current stage of the caregiving journey, the existence of other sources of strain, the caregivers' coping style and the density of their social network.[19] The consequences of informal caregiving also vary depending on the relationship between the care receiver and the caregiver, and on the acute or chronic nature of the care receiver's condition.[20]

Therefore, in designing caregiver interventions, it is essential to distinguish between elements that might be broadly applicable to all informal caregivers at large and elements that are specific to certain caregivers, care receivers' health problems and contexts of care.[21] Accordingly, a systematic review of 31 randomised controlled trials in the context of dementia and Alzheimer's disease found insufficient evidence to endorse the use of most interventions but noted larger trials that employed tailored interventions had significant effects on at least one outcome.[22] This exemplifies the limitation of taking a 'blanket approach' to designing interventions for caregivers at large, as these might fail to recognise significant effects on subsets of the caregiver population, thereby underestimating the wide heterogeneity in informal care provision and in the circumstances of caregivers.

Systematic reviews and meta-analyses of caregiving interventions often consider study populations as homogeneous, which masks contextual factors such as culture, financial resources, or health literacy that impact caregivers' risk of negative physical and mental outcomes.[23] This one-size-fits-all approach has been shown to be inadequate, but remains common in informal caregiving research.[24] We believe an umbrella review (i.e. a review of systematic reviews[25]) is a necessary step that will provide a broader picture of the effectiveness of different types of support services targeting different pools of informal caregivers. This requires combining an "aggregative approach", whereby quantitative reviews are used to answer well-defined questions based on empirical observations, together with more "configurative approaches" drawing not only on quantitative but also qualitative reviews.[26]

The aim of this umbrella review is to synthesise the evidence from existing systematic reviews evaluating the effectiveness of interventions to address the negative health consequences of informal caregiving. In particular, the following research questions will be addressed: 1) Are there effective interventions to prevent and reduce the negative health consequences of informal caregiving?; 2) Are certain types of interventions more effective than others?; 3) Is there evidence that the effectiveness of interventions depends on

For peer review only - http://bmjopen.bmj.com/site/about/guidelines.xhtml

caregiver, care receiver, care context and implementation characteristics?; and 4) How are the proposed interventions experienced by caregivers in terms of their acceptability, feasibility and added value?

To date, there are a few umbrella reviews or meta-reviews published on the topic of caregiver support. However, these reviews either include only one type of support intervention and/or disease,[27–29] or are too broad in nature in order to capture differences between caregiver groups.[30] This is the first umbrella review with a focus on caregivers of older populations, exploring objective physical and mental health-related outcomes and employing an integrative approach in order to complement quantitative effectiveness reviews with qualitative evidence on how caregivers experience support interventions.

METHODS AND ANALYSIS

Study design

We will conduct an umbrella review, defined as a ”review of existing systematic reviews and meta-analyses”.[25] Umbrella reviews focus on broad conditions or problems (e.g. interventions to mitigate the negative health consequences of informal caregiving) for which there is a rich and high-quality evidence base. The bird's-eye view provided by an umbrella review is well-suited to examine whether the evidence base around a given research question is consistent or contradictory, and to explore the underlying reasons in detail.[25] Furthermore, it will enable us to assess whether review authors who worked independently from each other but addressed similar research questions observed similar results and drew similar conclusions. This umbrella review will be reported in accordance with the Preferred Reporting Items for Overviews of Review (PRIOR) statement.[31] The review is anticipated to be conducted in the period of 1 April 2021 – 31 May 2022.

Target population

We will include systematic reviews of quantitative, qualitative, or mixed methods primary studies with or without meta-analysis focusing on interventions to address the negative health consequences of informal caregiving for informal caregivers of older adults. The full list of inclusion and exclusion criteria developed for this umbrella review is provided in **Table 1**.

For the purpose of this umbrella review, informal caregivers will be defined as any relatives, partners, friends or neighbours who have a significant personal relationship with, and provide a broad range of unpaid assistance for, older persons with a chronic or disabling condition.[32] Some have argued that the term 'family caregiver' should be preferred over 'informal caregiver' on the ground that caregivers may find the latter invalidating and belittling.[33] Although we acknowledge the importance of terminology around this complex issue, this umbrella review will use the term 'informal caregiving' to explicitly include non-familial caregivers in its scope. Older care receivers will be defined by chronological age or as having an age-related disease or syndrome such as dementia, cancer, stroke, Parkinson's disease, heart failure, multimorbidity and/or frailty. This corresponds with the broad range of caregiver experiences and needs that healthcare and social care providers tend to encounter. We will exclude reviews focusing exclusively on interventions directed towards care receivers, as well as those focusing on interventions implemented in the context of institutional care (addressing, for instance, the transition from home to care homes or the situation of disabled people ageing in residential facilities). Reviews of interventions specific to the context of end-of-life care will also be excluded, since caregiving approaches at this stage of the illness trajectory warrant a separate review and discussion.[34]

Interventions, comparators and outcomes

Systematic reviews will be included if they provide synthesised results about the effectiveness (i.e. the extent to which an intervention, when used appropriately, achieves the intended effect[35]) and implementation (i.e. how and why interventions work within real world conditions[36]) of interventions aimed at preventing and reducing negative health outcomes in the caregivers. We will examine all types of interventions (public or private), whether they are uni- or multi-faceted interventions, and delivered by all types of healthcare or social care professionals or volunteers. The typology developed by Gaugler et al[37]

For peer review only - http://bmjopen.bmj.com/site/about/guidelines.xhtml

will be adapted to categorise intervention types: skill building, psychosocial support, education, cognitive and behavioural approaches, respite, care/case management, and relaxation/physical activity.

In those reviews where a comparator is required, any comparator(s) tested will be considered: intervention versus control (no intervention, usual care, placebo or other control, or another intervention) as defined by the original reviews. We will include different caregiver-related health outcomes, since we want to give an overview of the different outcome measures used in the included reviews. This entails not only physical and mental diseases, syndromes, and symptoms, but also health-related quality of life and self-perceived health status. However, we will exclude reviews measuring exclusively non-health related outcomes, such as caregiver burden, family relations, work and financial status or stress/strain in the caregiver.

Publication type, date and language

Quantitative, qualitative, or mixed methods reviews published in a peer-reviewed journal or as a report done or mandated by an official health agency (e.g. Health Technology Assessment agency) will be included. Even if the first reviews on caregiving intervention research were published in the 1990s, we will focus on reviews published since 2000 in an attempt to capture studies taking place in the context of recent social changes and advancements in caregiving intervention research. We will restrict the inclusion to reviews published in languages spoken by research team members: English, Swedish, Spanish, French, Italian and German.

Data sources and search strategy

An iterative search strategy for bibliometric databases and grey literature will be developed to look for peer-reviewed systematic reviews evaluating interventions addressing the negative health consequences in the informal caregiver in all care settings, including quantitative and/or qualitative syntheses of implementation studies and process evaluations alongside trials testing such interventions. Therefore, the search for this umbrella review will aim to identify all research syntheses relevant to our four research questions. The search strategy will be guided by a librarian, comprehensively reported and the detailed search filters employed will be presented sequentially in the single appendix for all the databases that were searched and listed along with the search dates. The literature search will be undertaken using the following biomedical and sociological citation databases: Medline, CINAHL, PsycINFO and Web of Science. We will use *CoCites* (https://www.cocites.com/) citation-based search tool to widen the net and retrieve articles that cite eligible systematic reviews.[38]

The preliminary search strategy is detailed in In **Table 2**. This search strategy has been developed by the research team with the guidance of experienced health sciences librarians to estimate the number of potentially relevant systematic reviews. It combines free text words with controlled vocabulary terms (such as Medical Subject Headings (MeSH) terms/Thesaurus terms/CINAHL Subject Headings). Additional searches will be developed for syntheses of effectiveness, implementation or process evaluations published or mandated by official health agencies. We also expect a substantial number of such reports meeting our criteria not to be necessarily published in peer-reviewed journals. Thus, we will contact the first and last authors of selected reviews to retrieve grey literature that may otherwise have been missed. Finally, we will perform a manual search of the reference lists of relevant reviews.

Study selection

Titles and abstracts of the citations will be screened by two independent reviewers by using the Covidence (https://www.covidence.org/) software developed by the Cochrane collaboration.[39] Before screening, inclusion criteria will be tested in a pilot study. We will report any changes to the inclusion and exclusion criteria that result from the calibration exercise as deviations from the published protocol. Full texts of the relevant documents will be independently checked for eligibility by two independent reviewers using Covidence. Any dissent in abstract screening and full-text assessment will be resolved by discussion moderated by a third reviewer. Reviews excluded during the full-text assessment will be documented along with the reason for exclusion and presented using the Preferred Reporting Items for Systematic reviews and Meta-Analyses (PRISMA) flowchart.[40]

Methodological quality and risk of bias assessment

For peer review only - http://bmjopen.bmj.com/site/about/guidelines.xhtml

Critical appraisal of quantitative reviews will be conducted independently by two reviewers using the AMSTAR-2 checklist.[41] The checklist categorises the quality of the reviews based on seven critical and nine non-critical domains.[41] Based on the appraisal, the reviews will be grouped into critically low, low, moderate and high quality categories. Qualitative reviews will be assessed by two reviewers using an *ad hoc* quality appraisal checklist (**Appendix 1**), adapted from the Joanna Briggs Institute Checklist for Systematic Reviews and Research Syntheses.[25] The tool has been developed and piloted by all members of our multidisciplinary group. As far as possible, the quality of mixed-methods systematic reviews will be assessed using the above-mentioned tools for the quantitative and qualitative components, respectively. Any dissent in the process of quality assessment will be resolved through discussions moderated by a third reviewer.

Data extraction

Data will be extracted independently by two reviewers according to the type of systematic review (i.e. systematic reviews of quantitative, qualitative studies and/or mixed methods studies), using an *ad hoc* data extraction form that will be appended to the manuscript. This form will be calibrated during a pilot phase, with a purposive sample of five studies, to ensure that all relevant data are extracted in a consistent manner. Extraction will be limited to data reported in the reviews and will include broad categories of descriptive characteristics (such as number and list of primary studies, caregiver and care receiver demographics, context of care, type of interventions, etc.) and synthesised findings of the review (either meta-analysis or narrative findings). Clear indication of overlap of primary research studies in each of the included reviews will be presented, and significant overlap will be addressed to avoid double counting.

Data synthesis

A narrative description of the included reviews will be provided, with reference to a detailed table of included review characteristics. This description will allow for contextualising the results in terms of the relevance and evidence base of included research syntheses for the umbrella review questions. Specific items or points of interest from individual reviews will also be highlighted, as described below.

Overall effect estimates from quantitative systematic reviews will be presented in tables, alongside a detailed description of the interventions assessed. In addition to the direction and magnitude of effects, number of reviews that inform each outcome, number of participants, and statistical heterogeneity, factors related to the caregiver, care receiver and/or care context affecting the effectiveness of interventions will be identified whenever possible. This will attempt to answer research questions 1,2 and 3, related to effectiveness, comparison of interventions and factors affecting the effectiveness of interventions, respectively.

Evidence from syntheses of qualitative research will also be presented in tabular format. Results will be synthesised and illustrated by using verbatim replications from the source review when appropriate. Findings will also be described and interpreted in the research team's own words. Caregivers' experiences and views on barriers to and facilitators of interventions will be identified in terms of acceptability, feasibility and added value (research question 4). The diversity of caregivers, care receivers and/or care contexts will be considered as far as possible.

Framework synthesis methods will be employed for the integration of quantitative studies in effectiveness reviews with qualitative and process evaluation evidence. These methods are particularly suitable for capturing complexity of interventions in systematic reviews.[42]

An adapted version of Van Houtven et al's[43] framework will be used to organise caregiver interventions (**Figure 1**), which will be iteratively refined from the reports of completed reviews. As a complementary output, the final framework will be displayed in the form of diagrams showing the links between interventions' inputs and outputs.

Patient and public involvement (PPI)

For peer review only - http://bmjopen.bmj.com/site/about/guidelines.xhtml

No patients or members of the public were involved in the development of this umbrella review. However, the scope and methods of this review were informed by the literature and discussions with experts in the field.

Ethics and dissemination

The proposed umbrella review will rely exclusively on published data from secondary sources and will thus not involve any interactions with human subjects. It is therefore exempt from institutional review board (IRB) approval.

The results from the umbrella review will be presented at international congresses within the fields of e.g. gerontology and geriatrics, primary care, public health and social sciences, and will be published in a journal addressing a broad readership. Upon publication of the results, we will make the data generated by our research openly and publicly available. The team also intends to use media services available through the participating centres and funding organisation to publicise its findings via websites, social media and newsletters.

DISCUSSION

The majority of care received by the European older population is provided by informal family caregivers, and at least one third of them have extensive care needs that are unmet by social services.[44] The COVID-19 pandemic has further challenged social care for older people across the globe, unravelling its potential vulnerabilities. Informal caregivers are thus key agents in ensuring that community-living older people continue to experience a good quality of life. In the European context, this is further exacerbated by population aging, increasing levels of dependency, and cost containment.[1]

Caregiving is compatible with a low burden, but it can also exert a considerable cost on the physical and mental health of caregivers. This is especially true among older spouses caring for a highly dependent partner. Thus, if informal caregiving is to be sustainable, appropriate and timely, interventions need to prevent caregivers from suffering from its negative heath consequences, on top of the risk of health decline associated with their own age.

The synthesis of the evidence proposed in this umbrella review will provide directions to strengthen the resilience of social care system by supporting informal caregivers. An efficient coordination between viable formal and informal care will contribute to a better preparedness of the overall system of care for older people in dealing with potential future public health challenges.

For peer review only

CONTRIBUTORSHIP STATEMENT: All authors participated in conceptualising the methodology and planning the review process described in this protocol. AC-L drafted the protocol. MK, LD, LS and LM provided critical revisions and approved the final version of the protocol for submission. All authors agree to be accountable for all aspects of the work and in ensuring that questions related to the accuracy or integrity of any part of the work are appropriately investigated and resolved.

COMPETING INTERESTS STATEMENT: None declared.

FUNDING STATEMENT: This work was supported by the Swedish Research Council for Health, Working Life and Welfare (FORTE) grant number 2020-01544.

DATA SHARING STATEMENT: Data sharing is not applicable as no datasets were generated and/or analysed for this study.

ACKNOWLEDGMENTS: We wish to thank Emma-Lotta Säätelä and Jonas Pettersson, librarians at Karolinska Institutet, for preparing the search strategy and performing the searches across databases.

WORD COUNT: 2962

For peer review only

REFERENCES

1. Colombo F, Llena-Nozal A, Mercier J, Tjaden F. *Help Wanted? Providing and Paying for Long Term Care, OECD Health Policy Studies*. OECD Publishing; 2011. http://ec.europa.eu/health/reports/docs/oecd_helpwanted_en.pdf
2. Pavolini E, Ranci C. Restructuring the welfare state: Reforms in long-term care in Western European countries. *J Eur Soc Policy*. 2008;18(3):246-259. doi:10.1177/0958928708091058
3. Chan EYY, Gobat N, Kim JH, et al. Informal home care providers: the forgotten health-care workers during the COVID-19 pandemic. *Lancet*. 2020;395(10242):1957-1959. doi:10.1016/S0140-6736(20)31254-X
4. Verbeek-Oudijk D, Woittiez I, Eggink E, Putman L. Who cares in Europe? A comparison of long-term care fore the over-50s in sixteen European countries. *Hague Netherlands Inst Soc Res*. Published online 2014:1-109. http://www.google.nl/url?sa=t&rct=j&q=&esrc=s&source=web&cd=1&ved=0CCUQFjAA&url=http://www.scp.nl/dsresource?objectid=36940&type=org&ei=J5G_VNHoAoHeUOnpgoAC&usg=AFQjCNGLqTpygPklGLJ6Xsaat5OnvYyHtQ&sig2=X9Db9df5KLvp3NNslz-Gfg&bvm=bv.83829542
5. Broese van Groenou MI, De Boer A. Providing informal care in a changing society. *Eur J Ageing*. 2016;13(3):271-279. doi:10.1007/s10433-016-0370-7
6. Le Bihan B, Da Roit B, Sopadzhiyan A. The turn to optional familialism through the market: Long-term care, cash-for-care, and caregiving policies in Europe. In: *Social Policy and Administration*. Vol 53. ; 2019:579-595. doi:10.1111/spol.12505
7. Le Bihan B, Martin C. Diversification of care policy measures supporting older people: Towards greater flexibility for carers? *Eur J Ageing*. 2012;9(2):141-150. doi:10.1007/s10433-012-0228-6
8. Szebehely M, Trydegård GB. Home care for older people in Sweden: A universal model in transition. *Heal Soc Care Community*. 2012;20(3):300-309. doi:10.1111/j.1365-2524.2011.01046.x
9. Roth DL, Fredman L, Haley WE. Informal Caregiving and Its Impact on Health: A Reappraisal From Population-Based Studies. *Gerontologist*. 2015;55(2):309-319. doi:10.1093/geront/gnu177
10. Hiel L, Beenackers MA, Renders CM, Robroek SJW, Burdorf A, Croezen S. Providing personal informal care to older European adults: Should we care about the caregivers' health? *Prev Med (Baltim)*. 2015;70:64-68. doi:10.1016/j.ypmed.2014.10.028
11. Del-Pino-Casado R, Cardosa MR, López-Martínez C, Orgeta V. The association between subjective caregiver burden and depressive symptoms in carers of older relatives: A systematic review and meta-analysis. Cheungpasitporn W, ed. *PLoS One*. 2019;14(5):e0217648. doi:10.1371/journal.pone.0217648
12. Martín-Carrasco M, Martín MF, Valero CP, et al. Effectiveness of a psychoeducation intervention program in the reduction of caregiver burden in Alzheimer's Disease patients' caregivers. *Int J Geriatr Psychiatry*. 2009;24(5):489-499. doi:10.1002/gps.2142
13. Shaw C, McNamara R, Abrams K, et al. Systematic review of respite care in the frail elderly. *Health Technol Assess*. 2009;13(20 PG-1-224, iii):1-224, iii. doi:https://dx.doi.org/10.3310/hta13200
14. Haesler E, Bauer M, Nay R. Factors associated with constructive staff-family relationships in the care of older adults in the institutional setting. *JBI Libr Syst Rev*. 2006;4(9):1-72. doi:10.11124/jbisrir-2006-355
15. Fearing G, Sheppard CL, McDonald L, Beaulieu M, Hitzig SL. A systematic review on community-based interventions for elder abuse and neglect. *J Elder Abus Negl*. 2017;29(2-3):102-133. doi:10.1080/08946566.2017.1308286
16. Schulz R, Eden J. *Families Caring for an Aging America*. (Schulz R, Eden J, eds.). National Academies Press; 2016. doi:10.17226/23606
17. Williams F, Moghaddam N, Ramsden S, De Boos D. Interventions for reducing levels of burden amongst informal carers of persons with dementia in the community. A systematic review and meta-analysis of randomised controlled trials. *Aging Ment Heal*. 2019;23(12):1629-1642. doi:10.1080/13607863.2018.1515886
18. Pearlin LI, Mullan JT, Semple SJ, Skaff MM. Caregiving and the Stress Process: An Overview of Concepts and Their Measures. *Gerontologist*. 1990;30(5):583-594. doi:10.1093/geront/30.5.583
19. Mello J de A, Macq J, Van Durme T, et al. The determinants of informal caregivers' burden in the care of frail older persons: a dynamic and role-related perspective. *Aging Ment Heal*. 2017;21(8):838-843. doi:10.1080/13607863.2016.1168360
20. Conde-Sala JL, Garre-Olmo J, Turró-Garriga O, Vilalta-Franch J, López-Pousa S. Differential features of burden between spouse and adult-child caregivers of patients with Alzheimer's

For peer review only

disease: An exploratory comparative design. *Int J Nurs Stud*. 2010;47(10):1262-1273. doi:10.1016/j.ijnurstu.2010.03.001
21. Young HM, Bell JF, Whitney RL, et al. Social Determinants of Health: Underreported Heterogeneity in Systematic Reviews of Caregiver Interventions. Hepburn K, ed. *Gerontologist*. 2020;60(Supplement_1):S14-S28. doi:10.1093/geront/gnz148
22. Griffin J, Meis L, Greer N, et al. *Effectiveness of Family and Caregiver Interventions on Patient Outcomes Among Adults with Cancer or Memory-Related Disorders: A Systematic Review*.; 2013.
23. Bom J, Bakx P, Schut F, Van Doorslaer E. The Impact of Informal Caregiving for Older Adults on the Health of Various Types of Caregivers: A Systematic Review. *Gerontologist*. 2019;59(5):e629-e642. doi:10.1093/geront/gny137
24. Hepburn K, Siegel EO. New Directions to Advance Family Caregiving Research. *Gerontologist*. 2020;60(Supplement_1):S1-S4. doi:10.1093/geront/gnz182
25. Aromataris E, Fernandez R, Godfrey CM, Holly C, Khalil H, Tungpunkom P. Summarizing systematic reviews: Methodological development, conduct and reporting of an umbrella review approach. *Int J Evid Based Healthc*. 2015;13(3):132-140. doi:10.1097/XEB.0000000000000055
26. Petticrew M, Rehfuess E, Noyes J, et al. Synthesizing evidence on complex interventions: How meta-analytical, qualitative, and mixed-method approaches can contribute. *J Clin Epidemiol*. 2013;66(11):1230-1243. doi:10.1016/j.jclinepi.2013.06.005
27. Dickinson C, Dow J, Gibson G, Hayes L, Robalino S, Robinson L. Psychosocial intervention for carers of people with dementia: What components are most effective and when? A systematic review of systematic reviews. *Int Psychogeriatrics*. 2017;29(1):31-43. doi:10.1017/S1041610216001447
28. Cheng ST, Li KK, Losada A, et al. The effectiveness of nonpharmacological interventions for informal dementia caregivers: An updated systematic review and meta-analysis. *Psychol Aging*. 2020;35(1 PG-55-77):55-77. doi:https://dx.doi.org/10.1037/pag0000401
29. World Health Organization. Evidence profile: caregiver support. *Integr Care Older People Guidel Community-Level Interv to Manag Declines Intrinsic Capacit*. Published online 2017:35.
30. Dalton J, Thomas S, Harden M, Eastwood A, Parker G. Updated meta-review of evidence on support for carers. *J Heal Serv Res Policy*. 2018;23(3):196-207. doi:10.1177/1355819618766559
31. Pollock M, Fernandes RM, Pieper D, et al. Preferred Reporting Items for Overviews of Reviews (PRIOR): A protocol for development of a reporting guideline for overviews of reviews of healthcare interventions. *Syst Rev*. 2019;8(1):335. doi:10.1186/s13643-019-1252-9
32. U.S. Family Caregiver Alliance. Definitions. https://www.caregiver.org/resource/definitions-0/. Published 2021. Accessed March 10, 2021. https://www.caregiver.org/resource/definitions-0/
33. Stall NM, Campbell A, Reddy M, Rochon PA. Words Matter: The Language of Family Caregiving. *J Am Geriatr Soc*. 2019;67(10):2008-2010. doi:10.1111/jgs.15988
34. Penrod J, Hupcey JE, Baney BL, Loeb SJ. End-of-life caregiving trajectories. *Clin Nurs Res*. 2011;20(1):7-24. doi:10.1177/1054773810384852
35. Tufanaru C, Munn Z, Aromataris E, Campbell J HL. Chapter 3: Systematic reviews of effectiveness. In: *JBI Manual for Evidence Synthesis*. Joanna Briggs Institute; 2020. doi:https://doi.org/10.46658/JBIMES-20-04
36. Peters DH, Adam T, Alonge O, Agyepong IA, Tran N. Republished research: Implementation research: What it is and how to do it. *Br J Sports Med*. 2014;48(8):731-736. doi:10.1136/bmj.f6753
37. Gaugler JE, Jutkowitz E, Shippee TP, Brasure M. Consistency of dementia caregiver intervention classification: An evidence-based synthesis. *Int Psychogeriatrics*. 2017;29(1):19-30. doi:10.1017/S1041610216001514
38. Janssens ACJW, Gwinn M, Brockman JE, Powell K, Goodman M. Novel citation-based search method for scientific literature: a validation study. *BMC Med Res Methodol*. 2020;20(1):25. doi:10.1186/s12874-020-0907-5
39. Veritas Health Innovation. Covidence systematic review software. Published online 2021. www.covidence.org
40. Page MJ, McKenzie JE, Bossuyt PM, et al. The PRISMA 2020 statement: An updated guideline for reporting systematic reviews. *BMJ*. 2021;372. doi:10.1136/bmj.n71
41. Shea BJ, Reeves BC, Wells G, et al. AMSTAR 2: A critical appraisal tool for systematic reviews that include randomised or non-randomised studies of healthcare interventions, or both. *BMJ*. 2017;358:j4008. doi:10.1136/bmj.j4008
42. Harden A, Thomas J, Cargo M, et al. Cochrane Qualitative and Implementation Methods Group guidance series—paper 5: methods for integrating qualitative and implementation evidence within intervention effectiveness reviews. *J Clin Epidemiol*. 2018;97:70-78.

For peer review only - http://bmjopen.bmj.com/site/about/guidelines.xhtml

doi:10.1016/j.jclinepi.2017.11.029
43. Van Houtven CH, Voils CI, Weinberger M. An organizing framework for informal caregiver interventions: Detailing caregiving activities and caregiver and care recipient outcomes to optimize evaluation efforts. *BMC Geriatr*. 2011;11(1):77. doi:10.1186/1471-2318-11-77
44. Bień B, McKee KJ, Döhner H, et al. Disabled older people's use of health and social care services and their unmet care needs in six European countries. *Eur J Public Health*. 2013;23(6):1032-1038. doi:10.1093/eurpub/cks190

For peer review only

FIGURES

Figure 1. Conceptual framework linking caregiver interventions to caregiver health outcomes.

The lists within the boxes may not be fully exhaustive but are rather provided to present the specific research question driving the umbrella review.
[1]Typology inspired by the work by Gaugler et al.[37]
[2]An adapted version of Van Houtven et al's framework.[43]

For peer review only

Table 1. Inclusion and exclusion criteria for quantitative and qualitative reviews.

**Inclusion criteria**
Publication type, date and language<br>• Reviews published in a peer-reviewed journal OR as a report done or mandated by an official health agency (*e.g. Health Technology Assessment agency*)<br>• Reviews published between 1 January 2000, and 26 March 2021<br>• Reviews published in English, Swedish, Spanish, French, Italian or German
Study design<br>• For quantitative reviews: reviews including a reproducible, systematic search strategy, AND clearly defined inclusion/exclusion criteria AND risk of bias assessment for all included primary studies<br>• For qualitative reviews: reviews including a reproducible, systematic search strategy, AND defined inclusion/exclusion criteria
Population<br>• Reviews concerning informal caregivers (*i.e. people who regularly provide unpaid care to a family member, friend or neighbour*) of older people OR of persons presenting with aging-related disease (*e.g. dementia, stroke, Parkinson's disease, cancer, heart failure, multimorbidity, frailty*)
Intervention<br>• Reviews focusing on interventions and assessing either their effectiveness (for quantitative reviews) or their implementation and/or the lived experience of the target population (for qualitative reviews)
Outcome<br>• Reviews including physical or mental health-related outcomes of informal caregivers, including health-related quality of life
**Exclusion criteria**
• Reviews of interventions focusing *exclusively* on care receivers as the target population<br>• Reviews focusing *exclusively* on interventions for caregivers of young populations<br>• Reviews measuring *exclusively* non-health related outcomes, such as caregiver burden, stress/strain, work or financial status, family relations, breakdown of informal care<br>• Reviews focusing exclusively on end-of-life interventions or interventions implemented in the context of formal care institutions, including those addressing the transition from home to care homes

For peer review only

For peer review only - http://bmjopen.bmj.com/site/about/guidelines.xhtml

Table 2. Search strategy developed by the research team to estimate the sample size. Database(s): Ovid MEDLINE(R) and Epub Ahead of Print, In-Process, In-Data-Review & Other Non-Indexed Citations and Ovid MEDLINE(R) Daily 1946 to March 26, 2021.

#	Searches
1	exp Aged/
2	exp Aging/
3	Frailty/
4	(advanced age or aged or ageing or aging or elder* or frail* or geriatr* or gerontolog* or late* life or old age or old* adult* or old* client* or old* individual* or old* man or old* men or old* patient* or old* people or old* person* or old* population* or old* woman or old* women or oldest old or retired or senior*).ti,ab,kf.
5	Dementia/
6	Alzheimer Disease/
7	(dementia* or alzheimer*).ti,ab,kf.
8	exp Neoplasms/
9	(neoplasm* or cancer*).ti,ab,kf.
10	exp Stroke/
11	stroke.ti,ab,kf.
12	Parkinson disease/
13	parkinson*.ti,ab,kf.
14	Multimorbidity/
15	multimorbid*.ti,ab,kf.
16	exp Heart failure/
17	(heart failure or cardiac failure).ti,ab,kf.
18	or/1-17
19	Caregivers/
20	(caregiv* or care giv* or caretak* or care tak* or carer*).ti,ab,kf.
21	((family or informal or unpaid) adj3 (care or caring)).ti,ab,kf.
22	19 or 20 or 21
23	(meta analysis or systematic review).pt.
24	review.ti.
25	systematic* review*.ab,kf.
26	(meta analy* or metaanaly* or meta stud* or meta interpretation* or meta ethnograph* or meta summar* or meta synthes* or meta narrative* or mixed research synthes*).ti,ab,kf.
27	((concept analy* or grounded theory) and review*).ti,ab,kf.
28	or/23-27
29	18 and 22 and 28
30	(english or swedish or spanish or french or italian or german).lg.
31	29 and 30
32	limit 31 to yr="2000 -Current"
33	limit 32 to (comment or congress or editorial or letter)
34	32 not 33

Field labels: exp/ = exploded MeSH term; / = non exploded MeSH term; .ti,ab,kf. = title, abstract and author keywords; .pt. = publication type; adjx = within x words, regardless of order; * = truncation of word for alternate endings

For peer review only

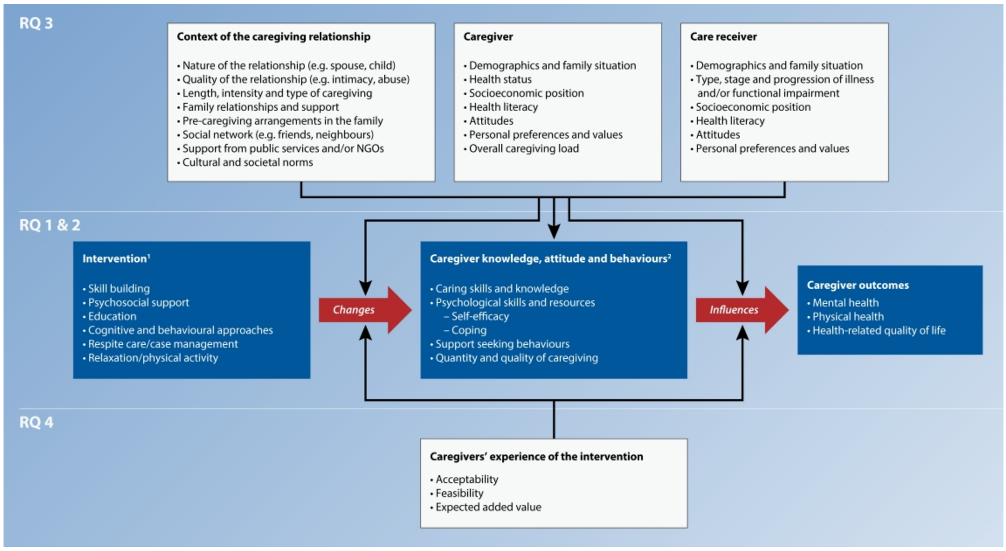

Figure 1. Conceptual framework linking caregiver interventions to caregiver health outcomes. The lists within the boxes may not be fully exhaustive but are rather provided to present the specific research question driving the umbrella review.

[1]Typology inspired by the work by Gaugler et al.[29]

[2]An adapted version of Van Houtven et al's framework.[34]

Appendix 1. Ad hoc quality appraisal checklist for qualitative reviews (adapted from Joanna Briggs Institute Checklist for Systematic Reviews and Research Syntheses[1]).

Questions	Possible responses
1. Was the research question clearly and explicitly stated?	Yes / No / Unclear / Not Applicable
2. Were the inclusion and exclusion criteria clearly described?	Yes / No / Unclear / Not Applicable
3. Was the literature search transparent and comprehensive enough to capture the relevant literature?	Yes / No / Unclear / Not Applicable
4. Did the authors use at least two databases for the literature search?	Yes / No / Unclear / Not Applicable
5. Did the authors perform a manual search of reference lists of included studies?	Yes / No / Unclear / Not Applicable
6. Was the study selection performed by two or more researchers independently?	Yes / No / Unclear / Not Applicable
7. Was the methodological quality formally assessed with explicit criteria?	Yes / No / Unclear / Not Applicable
8. Was the appraisal conducted by two or more researchers independently?	Yes / No / Unclear / Not Applicable
9. Was data extraction performed in duplicate or based on an established process of inter-author agreement assessment?	Yes / No / Unclear / Not Applicable
10. Were the synthesis methods justified?	Yes / No / Unclear / Not Applicable
11. Did the synthesis process involve two or more researchers?	Yes / No / Unclear / Not Applicable
12. Were the conclusions (including recommendations for policy and/or practice) clearly grounded in the results of the review?	Yes / No / Unclear / Not Applicable

For peer review only

REFERENCES

1. Aromataris E, Fernandez R, Godfrey CM, Holly C, Khalil H, Tungpunkom P. Summarizing systematic reviews: Methodological development, conduct and reporting of an umbrella review approach. *Int J Evid Based Healthc*. 2015;13(3):132-140. doi:10.1097/XEB.0000000000000055

For peer review only
